# A visualized and quercetin-optimized three-dimensional culture model of mouse ovaries derived from fetal gonads: A visualized and quercetin-optimized mouse ovarian 3D culture model

**DOI:** 10.3724/abbs.2025084

**Published:** 2025-06-03

**Authors:** Manman Cui, Ziye Zheng, Shiyu Bai, Zhaoxiang Ouyang, Jun Chen, Xinyan Yang, Cong Wan, Yi Zheng, Jiexiang Zhao, Gang Chang, Xiao-Yang Zhao

**Affiliations:** 1 State Key Laboratory of Organ Failure Research Department of Developmental Biology School of Basic Medical Sciences Southern Medical University Guangzhou 510515 China; 2 Department of Biochemistry and Molecular Biology Shenzhen University Medical School Shenzhen 518060 China; 3 Department of Gynecology Shunde Hospital Southern Medical University (The First People’s Hospital of Shunde Foshan) Foshan 528308 China; 4 The Seventh Affiliated Hospital Southern Medical University Foshan 528308 China; 5 Hainan Provincial Key Laboratory for Human Reproductive Medicine and Genetic Research & Key Laboratory of Reproductive Health Diseases Research and Translation Ministry of Education School of Basic Medicine and Life Sciences Hainan Medical University Haikou 571101 China; 6 State Key Laboratory of Organ Failure Research School of Laboratory Medicine and Biotechnology Southern Medical University Guangzhou 510515 China; 7 Guangdong Provincial Key Laboratory of Construction and Detection in Tissue Engineering Southern Medical University Guangzhou 510515 China; 8 Key Laboratory of Mental Health of the Ministry of Education Guangzhou 510515 China; 9 Guangdong-Hong Kong Joint Laboratory for Psychiatric Disorders Guangzhou 510515 China; 10 Department of Gynecology Zhujiang Hospital Southern Medical University Guangzhou 510515 China; 11 National Clinical Research Center for Kidney Disease Guangzhou 510515 China

**Keywords:** mouse ovarian explant 3D culture model, visualized platform, ROS, antioxidant, quercetin, modelling of ovarian diseases

## Abstract

The *in vitro* culture of ovarian tissue is emerging as a popular technology to study female reproductive medicine. However, standard *in vitro* culture conditions usually increase the level of reactive oxygen species (ROS), hindering ovarian development. Here, we establish an *in vitro* visualized mouse ovarian explant 3D culture model with the GFP-BVSC reporter system and obtain the early follicle pool from fetal female gonads. This model recapitulates *in vivo* ovarian characteristics and allows non-invasive monitoring of ovarian development. Importantly, supplementation with quercetin, a plant-derived natural antioxidant, increases the tissue area and total follicle count in cultured ovaries by protecting mitochondria and reducing ROS, thus more closely mimicking *in vivo* growth conditions. Finally, this visualized and optimized ovarian explant culture platform has been proven to be effective in modelling female ovarian diseases, such as the fetal reproductive aberrations of female offspring affected by gestational diabetes mellitus (GDM). Overall, our work extends the understanding of ovarian biology and creates an efficient and simplified platform for the morphological monitoring of ovarian development, as well as for drug screening and the clinical treatment of ovarian hypofunction.

## Introduction

On the basis of advancements in three-dimensional (3D) culture systems and increasing knowledge of ovary biology, many attempts have been made to establish ovarian organoid or ovarian explant culture models from fetal and adult ovaries, as well as pluripotent stem cells [ 1– 8] . Although this technology is still in its early stages, it can mimic the ovarian microenvironment and support follicle maturation, driving progress in reproductive and regenerative medicine [9]. Remarkably, Morohaku *et al*. [4] generated a complete ovarian explant model starting from mouse fetal female gonads and successfully obtained fertile pups. However, compared with that of germinal vesicle (GV) oocytes *in vivo*, the rate of embryonic development up to the pup stage remains to be further enhanced. Moreover, *in vitro* conditions inevitably lead to an increase in reactive oxygen species (ROS) generation from cellular metabolism or external factors [ 10, 11] . Excessive ROS can trigger a cascade of abnormal effects in the ovary, including apoptosis, inflammation, and mitochondrial damage, which collectively contribute to impaired ovarian development [ 12, 13] . Therefore, the real challenge is to reduce the impact of harmful exogenous factors on ovarian cells and simulate the growth system *in vivo* as much as possible. These findings will ultimately facilitate more precise studies of ovarian biology and disease pathology.

Ovarian dysfunction is a main cause of female infertility [14]. Approximately 1% of women under the age of 40 years experience primary ovarian insufficiency (POI), marked by a significant reduction in oocyte count and vitality, along with irregular menstrual cycles [15]. Germ cells in the ovary initiate meiosis around embryonic day 14.5 (E14.5) and then arrest at the diplotene stage of the first meiotic division from E17.5 to postnatal day 3 (PND3) in mice [16]. At this stage, primordial follicles begin to assemble, consisting of an arrested oocyte and a single layer of flattened granulosa cells [17]. Then, a cohort of primordial follicles are irreversibly activated and initiate folliculogenesis, and very few primordial follicles can ultimately develop into oocytes for fertilization, while many of them undergo degeneration [ 18, 19] . Notably, the primordial follicle pool represents the entire lifetime reserve of oocytes, and new primordial follicles cannot be produced after birth [ 20, 21] . Therefore, the yield and quality of the early primordial follicle pool determine a female’s reproductive health, laying the foundation for the delicate balance of fertility and hormonal regulation throughout life.

In recent years, a series of non-enzymatic antioxidants, known for their ability to reduce ROS, have been utilized to protect the female reproductive system, as well as the development of oocytes and embryos *in vitro* [ 10, 13, 22] . For example, melatonin can protect against cyclophosphamide (CTX)-induced POI and improve the quality of maternally aged oocytes by facilitating the relay of antioxidant metabolites [ 23, 24] . Coenzyme Q10 can increase antioxidant enzyme activity and decrease lipid peroxidation in cultured preantral follicles [25]. Moreover, it has been reported that flavonoid glycosides extracted from plants can facilitate ovarian development and have certain therapeutic effects on ovarian-related diseases, functioning as anti-inflammatory and antioxidant agents [ 26– 28] . Therefore, it is feasible to improve *in vitro* ovary culture conditions by using antioxidants.

In this study, we successfully established a mouse ovarian explant 3D model derived from the fetal female gonads of the BVSC transgenic strain, which provides a way to non-invasively track the development of ovaries *in vitro*. Moreover, antioxidants promoted the growth efficiency of the cultured ovaries, with quercetin demonstrating the most significant effect. Further studies revealed that quercetin had protective effects on mitochondria, reduced ROS accumulation and ultimately increased the number of follicles in the early follicle pool. Additionally, this visualized and optimized ovarian explant culture platform could serve as a valuable tool for establishing models of ovarian diseases, such as the fetal reproductive model of gestational diabetes mellitus (GDM)-affected F1 (the first filial) female offspring. Overall, we established a convenient ovarian explant culture platform with potential for ovarian disease modelling and drug screening.

## Materials and Methods

### Animals

All animal experiment procedures performed in this study were approved by the Ethics Committee on the Use and Care of Animals of Southern Medical University. The mice were maintained in accordance with the Ethical Guidelines of the Southern Medical University Ethics Committee (No. L2016149). Male BVSC transgenic mice [ *Blimp1*-mVenus and *Stella*-ECFP; membrane-targeted Venus (mVenus), enhanced cyan fluorescent protein (ECFP)] were generously provided by Mitinori Saitou Laboratory (Kyoto, Japan) [29], whereas female C57BL/6 mice were obtained from the Guangdong Medical Laboratory Animal Centre (Guangzhou, China). Female C57BL/6 mice in natural oestrus were mated with male BVSC transgenic mice. The GFP-BVSC reporter system is useful for visualizing ovarian development by marking germ cell populations [29]. At the early stages (E8.5–E14.5), *Blimp1*-mVenus and *Stella*-ECFP were co-expressed in primordial germ cells (PGCs) in both male and female gonads. From E15.5 onwards, *Stella*-ECFP expression was consistently observed in the germ cells of the female gonads. In neonatal ovaries, which contain oocytes at the follicle stage, *Stella*-ECFP expression was strongly reinitiated and persisted in developing oocytes. This spatiotemporal expression pattern of GFP-BVSC revealed the progression of female germ cell development.

All animals were housed under specific pathogen-free (SPF) conditions with a standard 12/12-h light/dark cycle and were provided unrestricted access to water and food. The ambient temperature was maintained at 20–25°C with a relative humidity of 40%–70%.

### Chemicals

ICI 182,780 (ICI; 100% purity; T2146) was purchased from TargetMol Chemicals (Shanghai, China) and was dissolved in DMSO to prepare a stock solution of 10 mM, and a final concentration of 10 μM was used in the experimental groups. Quercetin (98.45% purity; HY-18085) was purchased from MedChemExpress (Monmouth Junction, USA) and was dissolved in DMSO to prepare a stock solution of 10 mM, and a final concentration of 1 μM was used in the experimental groups. Glutathione (99.97% purity; S4606) was purchased from Selleck (Houston, USA) and was dissolved in H _2_O to prepare a stock solution of 160 mM, and a final concentration of 1 mM was used in the experimental groups. Vitamin C (98% purity; A4544) was purchased from Sigma (St Louis, USA) and was dissolved in H _2_O to prepare a stock solution of 100 mM, and a final concentration of 200 μM was used in the experimental groups. Ginsenoside Rg1 (99.74% purity; T2777) was purchased from TargetMol Chemicals and was dissolved in H _2_O to prepare a stock solution of 5 mM, and a final concentration of 1 μM was used in the experimental groups. Curcumin (98.98% purity; T1516) was purchased from TargetMol Chemicals and was dissolved in DMSO to prepare a stock solution of 20 mM, and a final concentration of 5 μM was used in the experimental groups. Melatonin (99.79% purity; T1659) was purchased from TargetMol Chemicals and was dissolved in DMSO to prepare a stock solution of 10 mM, and a final concentration of 0.1 μM was used in the experimental groups. Coenzyme Q10 (99.88% purity; T2796) was purchased from TargetMol Chemicals and was dissolved in DMSO to prepare a stock solution of 10 mM, and a final concentration of 5 μM was used in the experimental groups. Acetylcysteine (100% purity; T0875) was purchased from TargetMol Chemicals and was dissolved in H _2_O to prepare a stock solution of 100 mM, and a final concentration of 200 μM was used in the experimental groups. Metformin (100% purity; T8526) was purchased from TargetMol Chemicals and was dissolved in H _2_O to prepare a stock solution of 20 mM, and a final concentration of 50 μM was used in the experimental groups. Resveratrol (99.9% purity; T1558) was purchased from TargetMol Chemicals and was dissolved in H _2_O to prepare a stock solution of 10 mM, and a final concentration of 0.2 μM was used in the experimental groups. Vitamin B1 (99.05% purity; T0894) was purchased from TargetMol Chemicals and was dissolved in H _2_O to prepare a stock solution of 10 mM, and a final concentration of 10 μM was used in the experimental groups. All the compounds were stored at –80°C for long-term use.

### Isolation and culture of female gonad organs from E12.5 mouse fetuses

After mating, the female mice were checked, and the appearance of a vaginal plug was defined as embryonic day 0.5 (E0.5). At E12.5, female gonad organs were identified on the basis of their dispersed morphology. The isolated female gonad organs, excluding the mesonephros, were cultured in 1% agar blocks (10 mm ×10 mm) in 24-well tissue culture plates at 37°C in humidified incubators with 5% CO _2_. As previously described [4], the culture medium consisted of α-MEM (PM150421; Pricella, Wuhan, China) supplemented with 10% fetal bovine serum (FBS; SE100-011; VISTECH, Beijing, China) and 1% penicillin-streptomycin. The estrogen receptor antagonist ICI 182,780 was added from day 5 to day 11 at a concentration of 10 μM. In the study by Morohaku *et al*. [4], elevated estrogen signaling—due to certain ligands in FBS—was found to result in hypoplastic follicle formation *in vitro*. To eliminate this effect, the addition of ICI 182,780, an estrogen receptor antagonist, during the initial stage of *in vitro* folliculogenesis (from day 5 to day 11) led to an increased number of single secondary follicles compared with the original conditions. Furthermore, ICI 182,780 treatment promoted the formation of a complete laminin envelope around individual follicles, enhancing follicular structure and development.

The medium in each well was replaced by fresh medium every other day. The cultured ovaries at the developmental stage of interest were photographed via a DMi8 inverted fluorescence microscope (Leica, Wetzlar, Germany).

### Immunofluorescence staining

The ovaries both *in vivo* and *in vitro* were fixed in 4% paraformaldehyde at 4°C overnight. After fixation, the samples were dehydrated, embedded in paraffin, and sectioned into 5-μm-thick slices. The sections were deparaffinized, rehydrated, and subjected to antigen retrieval by boiling in EDTA buffer (pH 8.0). Following antigen retrieval, the sections were cooled to room temperature and blocked with buffer containing 2% bovine serum albumin (BSA) in PBS for 1 h. Primary antibodies were then applied to the sections, which were subsequently incubated in a humidified chamber at 4°C overnight. Following this, the sections were incubated with secondary antibodies for 1 h at room temperature. The nuclei were stained with Hoechst 33342 for 15 min at room temperature. Finally, images were captured via an LSM880 confocal microscope (Zeiss, Oberkochen, Germany).

The primary antibodies used were as follows: mouse anti-DDX4 (1:500, ab27591; Abcam, Cambridge, UK), rabbit anti-DDX4 (1:500, ab13840; Abcam), rabbit anti-FOXL2 (1:800, ab246511; Abcam), rabbit anti-STRA8 (1:500, ab49602; Abcam), mouse anti-SYCP3 (1:500, ab97672; Abcam), and rabbit anti-cleaved PARP1 (cPARP1) (1:500, ab32064; Abcam). The secondary antibodies used were as follows: Alexa Fluor 488-conjugated goat anti-rabbit IgG (1:500, #111-545-003; Jackson ImmunoResearch, West Grove, USA), Alexa Fluor 594-conjugated goat anti-rabbit IgG (1:500, #111-585-003; Jackson ImmunoResearch), Alexa Fluor 488-conjugated goat anti-mouse IgG (1:500, #115-545-003; Jackson ImmunoResearch), and Alexa Fluor 594-conjugated goat anti-mouse IgG (1:500, #115-585-003; Jackson ImmunoResearch).

### Western blot analysis

The ovarian cells were lysed in cold RIPA buffer (R0010; Solarbio, Beijing, China) supplemented with 1 mM protease inhibitor cocktail (C0001; TargetMol Chemicals) for 30 min. The lysates were centrifuged at 12,000 *g* for 15 min, and the supernatants were collected for further use. Protein samples were separated by SDS-PAGE and transferred onto nitrocellulose membranes. The following primary antibodies were used in this study: rabbit anti-FASN (1:1000, 3180T; CST, Danvers, USA), rabbit anti-HK2 (1:500, A0994; ABclonal, Wuhan, China), rabbit anti-TNFα (1:1000, HA722022; HUABIO, Hangzhou, China), rabbit anti-Caspase-3 (1:500, T40044F; Abmart, Shanghai, China), and rabbit anti-β-actin (1:5000, AC038; ABclonal). The secondary antibody used in this study was HRP-conjugated goat anti-rabbit IgG (1:5000, SA00001-2; Proteintech, Rosemont, USA). An enhanced chemiluminescence (ECL) kit (36208ES60; YEASON, Shenzhen, China) was used on the membrane before film exposure.

### Detection of ROS levels

To measure the levels of intracellular ROS, fresh ovarian single-cell suspensions or whole ovarian tissues from each group were collected and subsequently incubated for 30 min at 37°C in α-MEM supplemented with 10 mM dihydroethidium (DHE; D807594; Macklin, Shanghai, China) in the dark. After being washed with PBS, the ovarian cells and ovarian tissues were imaged under an inverted fluorescence microscope with consistent parameters, and the ovarian cells were detected via a flow cytometer (CytoFlex; Beckman, Indianapolis, USA). The fluorescence intensity of the ROS in each group was quantified with ImageJ software (NIH, Bethesda, USA).

### Measurement of cell apoptosis rate

To measure the rate of cell apoptosis in ovaries both *in vivo* and *in vitro*, immunofluorescence images were analyzed using ImageJ software. The images were converted into 8-bit format. Positive cells for each marker were manually counted via the “multipoint” tool. DDX4 ^+^ cells represented all germ cells/oocytes, whereas DDX4 ^+^ and cPARP1 ^+^ cells represented germ cells/oocytes in a state of cell apoptosis. DDX4 ^–^ cells represented all somatic cells, whereas DDX4 ^–^ and cPARP1 ^+^ cells represented somatic cells in a state of cell apoptosis.

### Quantification of ovarian follicles

Briefly, immunofluorescence for DDX4 (a marker of oocytes) and FOXL2 (a marker of granulosa cells) was performed to identify the morphology of the follicles. Every fifth section of the entire ovary was photographed for follicle counting. Follicles at different stages of development, including primordial follicles (an oocyte surrounded by one layer of flattened granulosa cells), primary follicles (an oocyte surrounded by one layer of cuboidal granulosa cells), and secondary follicles (more than one layer of cuboidal granulosa cells with no antral space), were scored in each section, according to a previous report [30]. The cumulative follicle count was multiplied by a correction factor of 5 to represent the estimated number of total follicles in an ovary [31].

### RNA extraction and qPCR analysis

Total RNA was extracted using TRIzol Reagent (Y1809; TIANGEN, Beijing, China). According to the manufacturer’s instructions, reverse transcription was performed with HiScript QRT SuperMix for qPCR (R123-01; Vazyme, Shanghai, China), and qPCR was conducted using the AceQ qPCR SYBR Green Master Mix (Q121-02; Vazyme). The expression levels of the target genes were normalized to the expression of *Gapdh* and were calculated via the comparative cycle threshold method (2 ^–ΔΔCt^). All the qPCR primer pairs used in this study are listed in Supplementary Table S1.

### RNA-seq library construction and sequencing

Total RNA was extracted via TRIzol Reagent. Messenger RNA was purified from total RNA using poly-T oligo-attached magnetic beads. After fragmentation, first-strand cDNA was synthesized using random hexamer primers, followed by second-strand cDNA synthesis. The library was ready after end repair, A-tailing, adapter ligation, size selection, amplification, and purification. After library quality control, the quantified libraries were pooled and sequenced on the Illumina platform (Illumina, San Diego, USA) according to the effective library concentration and data amount.

### Quality control of RNA-seq data

The original fluorescence image files obtained from the Illumina platform were transformed to short reads (Raw data) via base calling, and these short reads were recorded in FASTQ format, which contains sequence information and corresponding sequencing quality information. Fastp (version 0.23.1) was subsequently used to perform basic statistics on the quality of the raw reads [32]: (1) Discard paired reads if one read contains adapter contamination; (2) Discard paired reads if more than 10% of bases are uncertain in one read; and (3) Discard paired reads if the proportion of low-quality (Phred quality < 5) bases is greater than 50% in one read.

### Differentially expressed gene analysis

The clean reads obtained after quality control were aligned to the reference genome using HISAT2 (version 2.2.1) [33]. The featureCounts software (version 2.0.1) was then used to count the number of reads mapped to each gene, producing a gene expression matrix [34]. Normalization of the gene expression levels was performed using the trimmed mean of M values (TMM) method in the edgeR package, ensuring comparability across samples. Differential expression analysis between groups was conducted via the DESeq2 package (version 1.34.0) [35]. Gene Ontology (GO) analysis was performed via the Metascape tool. All analyses were carried out in R (version 4.1.2).

### Gene set enrichment analysis

Gene set enrichment analysis (GSEA) was used to identify gene sets that were significantly different between the control and quercetin groups. In brief, we used the gseKEGG function to perform GSEA with the clusterProfiler R package [36] under the default setting.

### Measurement of GSH and NADH

The intracellular levels of GSH and NADH were individually measured in ovarian cell lysates using the GSH and GSSG Assay Kit (#S0053; Beyotime, Shanghai, China) and the NAD ^+^/NADH Assay Kit with WST-8 (#S0175; Beyotime).

### Quantification of the mitochondrial membrane potential

Tetramethylrhodamine ethyl ester (TMRE) (ENZ-52309; Enzo Life Sciences, Beijing, China) was used to measure the mitochondrial membrane potential (MMP). The ovaries both *in vivo* and *in vitro* were trypsinized with 0.25% trypsin/EDTA (Gibco, Carlsbad, USA), pelleted at 300 *g*, and resuspended in 200 μL of fresh α-MEM supplemented with 50 nM TMRE. The samples were then incubated at 37°C in the dark for 20 min. After being washed with PBS, the samples were analyzed with a flow cytometer (CytoFlex). The mean fluorescence intensities (MFIs) of the three replicates were calculated for each sample, and the MFI values *in vitro* with DMSO or quercetin were compared to those *in vivo*.

### Measurement of ATP levels

An Enhanced ATP Assay Kit (S0027; Beyotime) was used to determine ATP levels. Briefly, the ovaries both *in vivo* and *in vitro* were trypsinized with 0.25% trypsin/EDTA, pelleted at 300 *g*, and resuspended in 100 μL of lysis buffer. After lysis, the mixture was centrifuged at 12000 *g* for 5 min at 4°C, and the supernatant was pipetted for subsequent determination with a luminometer. For each assay, a five-point standard curve (0.001, 0.01, 0.1, 1, and 10 μM ATP) was created, and the ATP content was determined using the formula obtained from the linear regression of the standard curve.

### Measurement of mitochondrial morphology

MitoTracker® Red FM (M22425; Thermo Fisher Scientific, Waltham, USA) was used to measure mitochondrial morphology. Ovaries were digested into a single-cell suspension and resuspended in 200 μL of fresh PBS (with 2% FBS) supplemented with 50 nM or 150 nM MitoTracker. The samples were then incubated at 37°C in the dark for 20 min. After being washed with PBS, the samples stained with 50 nM MitoTracker were analyzed by flow cytometry, whereas the samples stained with 150 nM MitoTracker were analyzed using a Zeiss LSM880 confocal microscope.

### Measurement of glucose and triglyceride levels

Ovarian culture supernatants were collected after 32 h of culture. The samples were then centrifuged at 3000 *g* for 15 min at 4°C to collect the supernatant. A fully automatic biochemical analyzer (Chemray 800; Rayto Life and Analytical Sciences Co., Ltd., Shenzhen, China) was used to assess the biochemical parameters of the supernatant. Biochemical kits for glucose (GLU; S03039) and triglycerides (TG; S03027) were purchased from Rayto (Shenzhen, China).

### Cell death analysis

Annexin V-FITC/PI Apoptosis Detection Kit (C1062S; Beyotime) was used to determine cell death in ovaries cultured under control and high glucose conditions. Briefly, ovaries were enzymatically digested into a single-cell suspension and resuspended in 200 μL of binding buffer containing Annexin V-FITC and propidium iodide (PI). The samples were then incubated in the dark at room temperature for 20 min. Following incubation, the cells were washed with PBS and analyzed using a flow cytometer.

### Statistical analysis

All the experiments were repeated at least two times. Statistical analysis was performed using GraphPad Prism (version 6.0.0). The significance of differences between the two groups was analyzed by unpaired two-sided Student’s *t* test. The significance of differences among three or more groups was analyzed by one-way ANOVA. Data are expressed as the mean ± SD. A *P* value of less than 0.05 was considered to indicate a significant difference.

## Results

### *In vitro* reproduction of primordial follicle pool formation in gonad-derived ovaries

**Figure 1:**
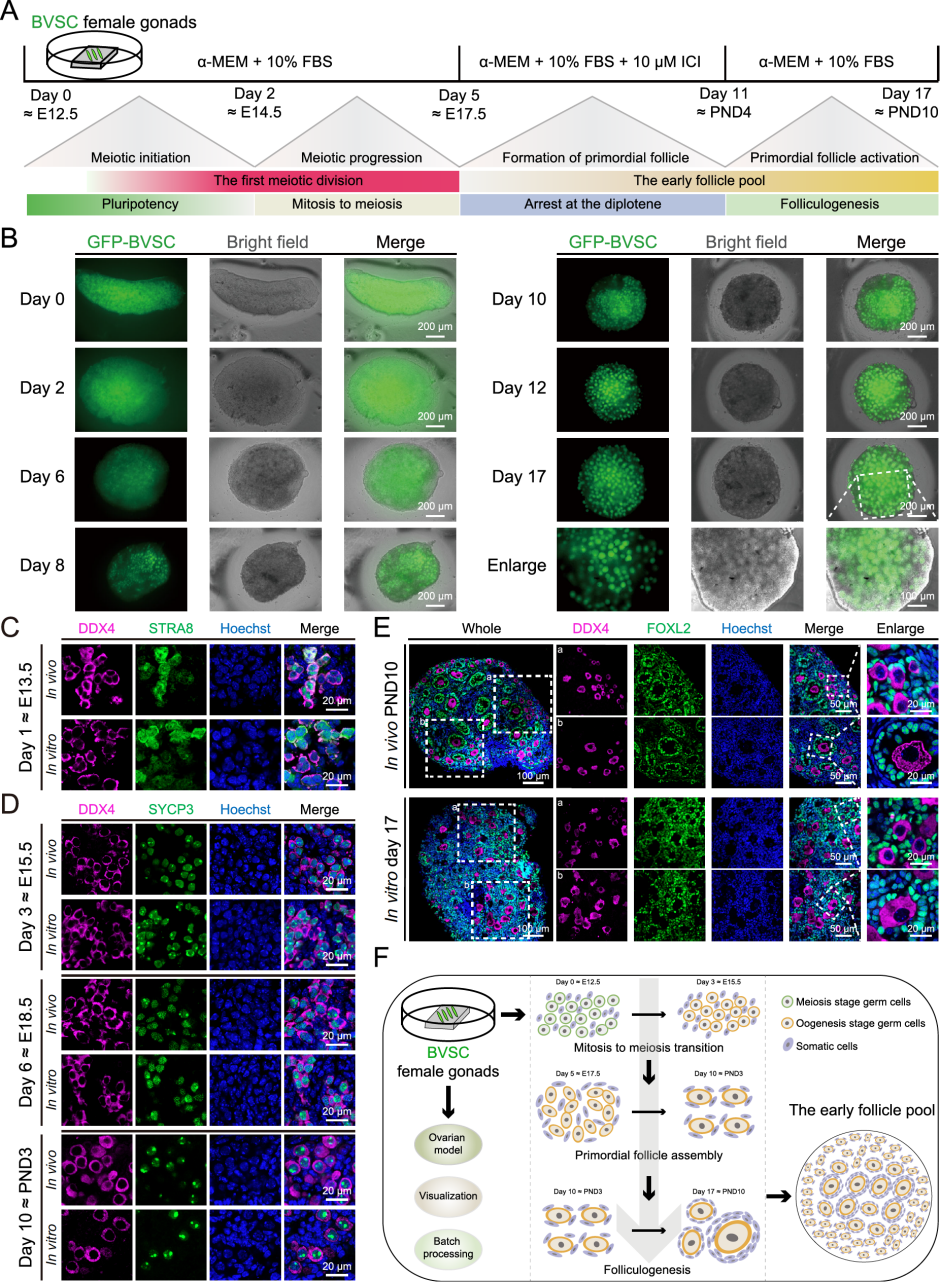
Figure 1 A visualized mouse ovarian explant 3D culture model derived from fetal female gonads using the *Blimp1*-mVenus and *Stella*-ECFP transgenic strains (A) Schematic overview of the milestones in the development of ovaries both in vivo and in vitro. (B) Representative images of GFP-BVSC fluorescence (green) and bright field (gray) images of cultured ovaries at the indicated time points. Scale bar, 200 μm (top) and 100 μm (bottom). (C) Immunostaining of STRA8 (green) co-stained with DDX4 (purple) and Hoechst (blue) in ovaries both in vivo and in vitro. Scale bar, 20 μm. (D) Immunostaining of SYCP3 (green) co-stained with DDX4 (purple) and Hoechst (blue) in ovaries both in vivo and in vitro. Scale bar, 20 μm. (E) Immunofluorescence of DDX4 (purple) co-stained with FOXL2 (green) and Hoechst (blue) in PND10 ovaries in vivo and day 17 in vitro. Scale bar, 100 μm (left); 50 μm (middle); 20 μm (right). (F) Summary diagram of the progressive growth of ovaries in vitro.

*In vitro* reconstitution of oogenesis is a key question in the field of reproductive biology and regenerative medicine; therefore, the culture of ovarian explants has great implications for both female reproductive medicine and research [ 37, 38] . Moreover, the culture of ovarian explants allows researchers to conduct more studies utilizing transgenic mice. The previously established *Blimp1*-mVenus and *Stella*-ECFP (BVSC) transgenic strain facilitates the comprehensive detection of the female germ cell lineage, thus providing a valuable tool not only for the continuous monitoring of germ cell development and follicular assembly but also for an in-depth exploration of their regulatory mechanisms both *in vivo* and *in vitro* [ 6, 29, 39– 41] . In this study, female gonads from BVSC embryos at E12.5 were obtained and cultured *in vitro* for 17 days on a 1% agarose gel in α-MEM supplemented with 10% FBS. From day 5 to day 11, the medium was treated with 10 μM estrogen receptor antagonist ICI, which corresponded to the period of primordial follicle formation *in vivo* ( Figure 1A). Through the acquisition of sequential images of GFP-BVSC fluorescence at multiple time points, we systematically documented the comprehensive developmental process of ovaries *in vitro*. Generally, cultured ovaries underwent a morphological transition from a long ribbon-shaped configuration to a spherical configuration, closely mirroring the developmental characteristics observed *in vivo* [29]. Specifically, GFP-BVSC fluorescence confirmed the presence of germ cells/oocytes, and a single follicle structure containing GFP-BVSC-positive (BVSC ^+^) oocytes emerged within the time frame spanning from day 10 to day 17 ( Figure 1B).

Notably, we confirmed that germ cells within cultured ovaries could initiate meiosis in a manner highly similar to that *in vivo*, as judged by the expression and localization patterns of STRA8 (stimulated by retinoic acid gene 8) and SYCP3 (synaptonemal complex protein 3) ( Figure 1C,D and Supplementary Figure S1A–C). Histologically, small primordial follicles and large secondary follicles were marked by DDX4 and FOXL2 in the ovaries cultured on day 17, resembling those observed *in vivo* on PND10 ( Figure 1E). Moreover, this model demonstrated significant potential for conducting experiments in batches. For example, eight pairs of female gonads could be obtained from one pregnant mouse, and they could be randomly allocated to eight groups (approximately two gonads per group), enabling the performance of batch experiments ( Supplementary Figure S1D). Additionally, we further segmented the female gonads into small pieces (approximately 1/2 to 1/3 of the original size) and successfully obtained the early follicle pool ( Supplementary Figure S1E). We found that the number of BVSC ^+^ cells was positively correlated with the area of ovarian tissue, indicating the uniformity of tissue development in culture ( Supplementary Figure S1F). On the basis of these results, the experimental throughput of the ovarian explant 3D model could be significantly increased, thereby broadening its potential applications in disease modelling and drug screening.

In summary, we successfully developed an *in vitro* visualized ovarian explant 3D culture model for obtaining the early follicle pool derived from fetal female gonads with the aid of the GFP-BVSC reporter system, facilitating morphological monitoring and cell sorting, as well as batch processing ( Figure 1F). In the subsequent studies of this work, we continued to optimize this system and explore its potential applications, providing key insights into the functional evolution of ovaries and shedding light on underlying biological processes.

### Elevated oxidative stress leads to increased cell apoptosis in cultured ovaries

**Figure 2:**
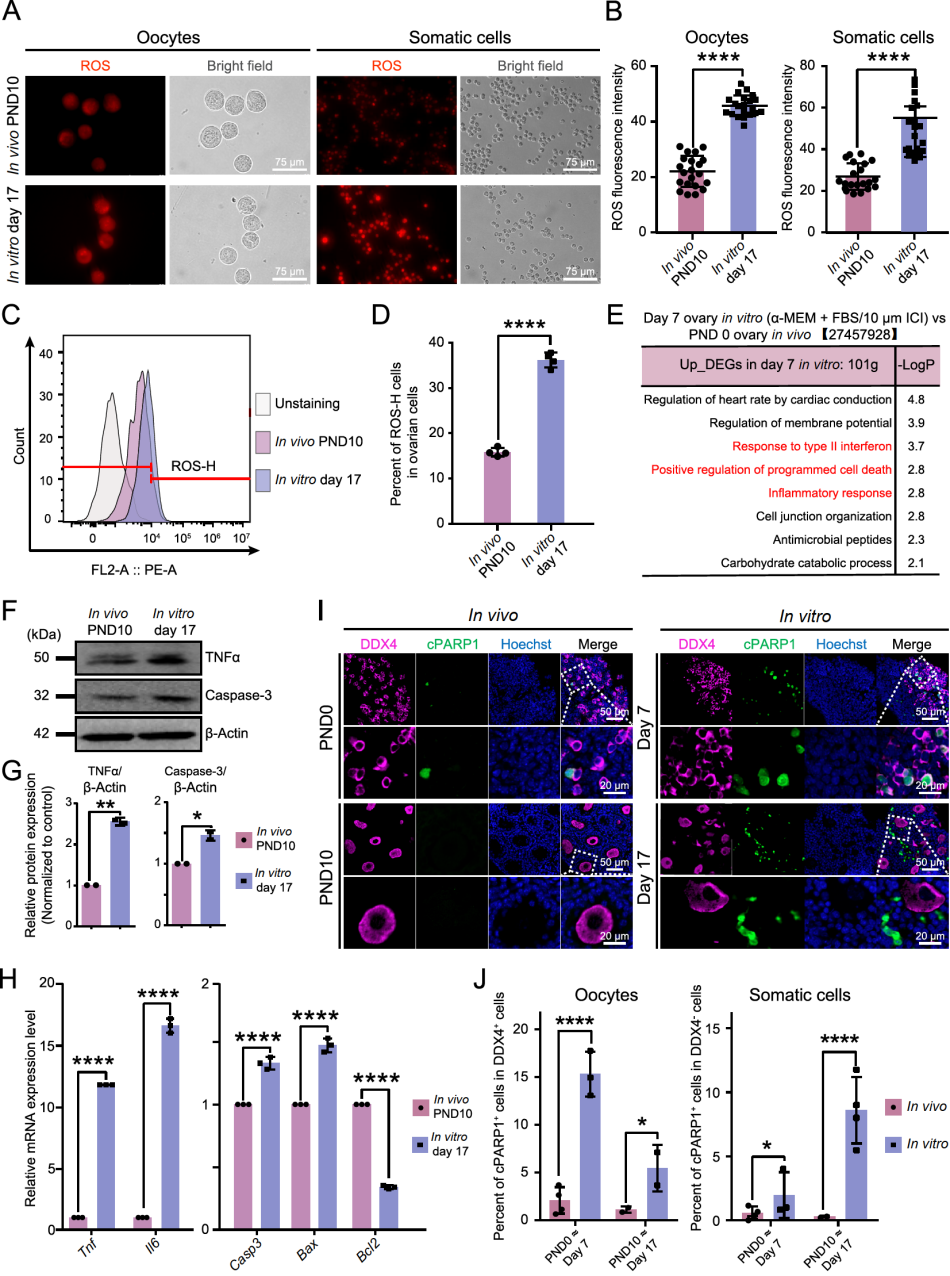
Figure 2 Elevated ROS and cell apoptosis in cultured ovaries (A) Representative images of ROS fluorescence (red) and bright field (gray) images of oocytes (left) and somatic cells (right) at PND10 in vivo and day 17 in vitro. The cells were imaged under an inverted fluorescence microscope via consistent parameters. Scale bar, 75 μm. (B) Quantitative analysis of the ROS fluorescence intensity of oocytes (left) and somatic cells (right) in Figure 2A. Data are presented as the mean ± SD with data points (21 and 21 oocytes and 21 and 21 somatic cells; n = 2 biologically independent samples); unpaired two-sided Student’s t test; ****P < 0.0001. (C) Flow cytometry analysis of intracellular ROS in PND10 ovaries in vivo and day 17 ovaries in vitro through staining with dihydroethidium. PE-A+ cells are indicators of ROS-H cells. (D) Quantitative analysis of the ROS-H cells in Figure 2C. Data are presented as the mean ± SD (n = 4 biologically independent samples); unpaired two-sided Student’s t test; ****P < 0.0001. (E) Enriched GO terms (performed via the Metascape tool with a well-adopted hypergeometric test and Benjamini-Hochberg p value) of the upregulated genes in day 7 ovaries cultured in vitro. (F) Immunoblots of the indicated proteins in PND10 ovaries in vivo and day 17 in vitro. (G) The relative expression levels of the proteins in Figure 2F; unpaired two-sided Student’s t test; *P < 0.05, **P < 0.01. (H) qPCR analysis of the expression of marker genes in PND10 ovaries in vivo and day 17 in vitro. Data are presented as the mean ± SD (n = 3 biologically independent samples); unpaired two-sided Student’s t test; ****P < 0.0001. (I) Immunostaining of cPARP1 (green) co-stained with DDX4 (purple) and Hoechst (blue) in ovaries both in vivo and in vitro at the indicated time points. Scale bar, 50 μm (top) and 20 μm (bottom). (J) Quantitative analysis of the percentage of cPARP1+ cells among DDX4+ oocytes (left) and DDX4– somatic cells (right) at the indicated time points in Figure 2I. Data are presented as the mean ± SD (n ≥ 2 biologically independent samples); unpaired two-sided Student’s t test; *P < 0.05, ****P < 0.0001.

Previous studies have indicated that excessive ROS are inevitably generated from endogenous and exogenous sources, such as light and general physicochemical parameters, in culture systems [ 11, 42, 43] . The accumulation of ROS induces a cascade of subsequent reactions and eventually results in cell damage [ 44, 45] . We speculated that there was also an abnormal increase in ROS in the ovarian explant culture system, which might be one of the important factors affecting the size of the ovarian follicle pool. To determine the cellular redox (reduction and oxidation) balance in cultured ovaries *in vitro* compared with that *in vivo*, we conducted further analyses. In detail, there was a significant increase in intracellular ROS in both oocytes and somatic cells in the ovaries cultured on day 17, in contrast with those observed *in vivo* on PND10 ( Figure 2A,B). Flow cytometry also revealed a global increase in ROS levels in the cultured ovaries ( Figure 2C,D).

Excessive ROS can lead to oxidative stress or overstimulate inflammatory responses, leading to damage to DNA and proteins and potentially resulting in cell apoptosis and tissue damage [ 22, 46] . We reanalyzed the RNA-seq data reported by Morohaku *et al*. [4] and found that, compared with *in vivo* ovarian cells, *in vitro* cultured ovarian cells presented increased expression of genes associated with “positive regulation of programmed cell death” and “inflammatory response” ( Figure 2E). Therefore, we examined the expression levels of key genes involved in the relevant pathways. The western blot analysis and qPCR results indicated that *in vitro*, the expressions of inflammation- and apoptosis-related factors, such as TNFα (a key proinflammatory cytokine) and Caspase-3 (a key mediator of cell apoptosis), were increased in the ovaries ( Figure 2F–H). Moreover, *Bax* (a proapoptotic gene) was upregulated in cultured ovaries, accompanied by the downregulation of *Bcl2* (an antiapoptotic gene) ( Figure 2H). The cultured ovaries presented a significantly greater proportion of cells positive for cleaved PARP1 (cPARP1 ^+^), a marker of cell apoptosis, particularly in oocytes on day 7 and in somatic cells on day 17 *in vitro* ( Figure 2I,J).

Collectively, these findings indicated that in cultured ovaries, aggravated oxidative stress resulted in a significant increase in cell apoptosis in both oocytes and somatic cells.

### Optimizing the ovarian explant 3D culture model by screening antioxidants against ROS

**Figure 3:**
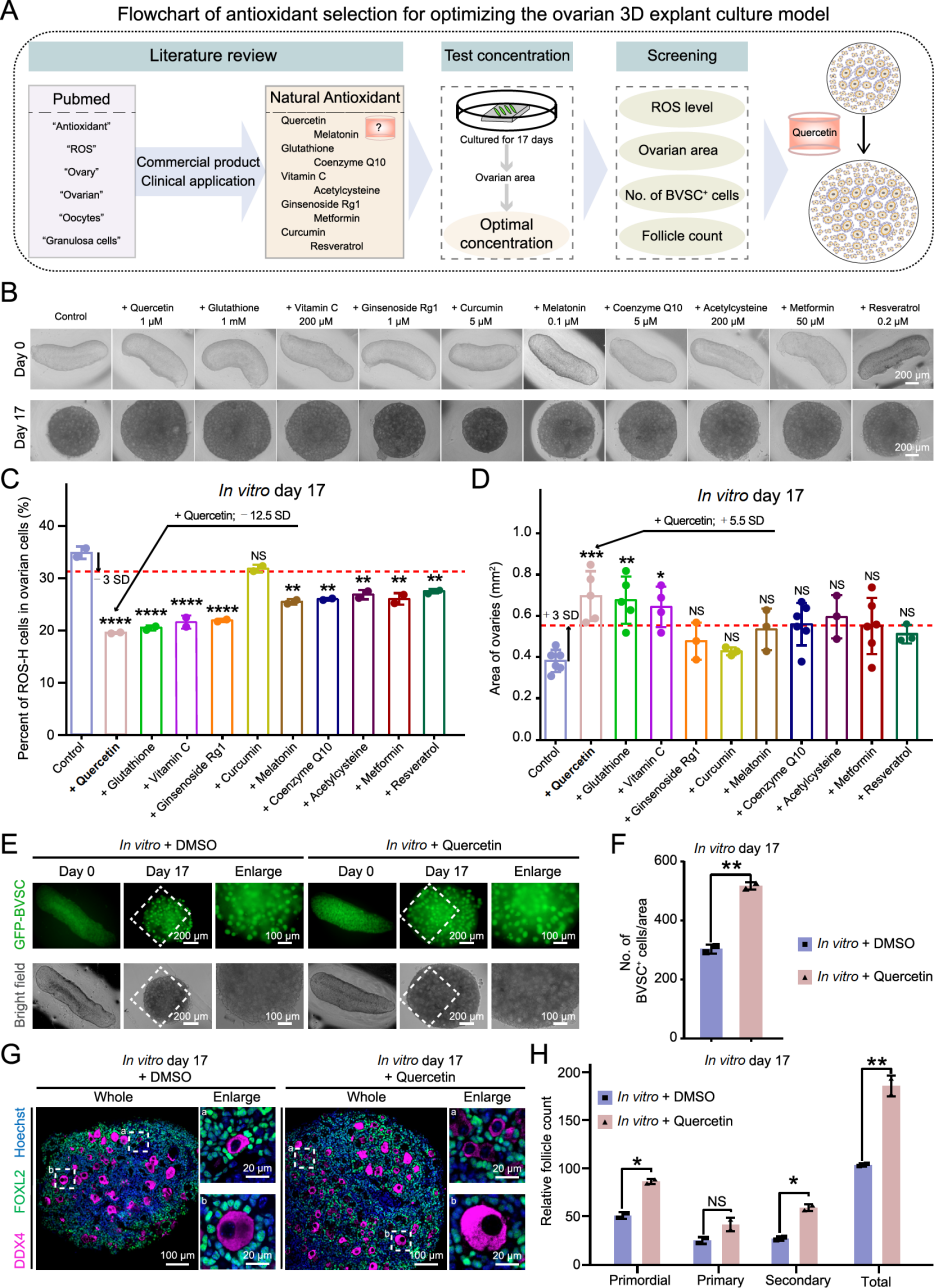
Figure 3 Selection of antioxidants against ROS in cultured ovaries (A) Flowchart of antioxidant selection for optimizing the ovarian explant 3D culture model. (B) Bright field images of ovaries in various culture media at the indicated time. Scale bar, 200 μm. (C) Quantitative analysis of ROS-H cells in various culture media from ovaries cultured on day 17, corresponding to Figure 3B. The red dotted lines indicate the -3 SD of the control group. Data are presented as the mean ± SD (n = 2 biologically independent samples); one-way ANOVA; NS, not significant; **P < 0.01; ****P < 0.0001. (D) Quantitative analysis of the ovarian area in various culture media in the ovaries cultured on day 17, corresponding to Figure 3B. The red dotted lines indicate the + 3 SD of the control group. Data are presented as the mean ± SD (n ≥2 biologically independent samples); one-way ANOVA; NS, not significant; *P < 0.05, **P < 0.01, ***P < 0.001. (E) Representative images of GFP-BVSC fluorescence (green) and bright field (gray) images of ovaries in vitro at the indicated time points. Scale bar, 200 μm (left); 100 μm (right). (F) Quantitative analysis of the number of BVSC+ cells in the ovaries cultured on day 17, corresponding to Figure 3E. Data are presented as the mean ± SD (n = 2 biologically independent samples); unpaired two-sided Student’s t test; **P < 0.01. (G) Immunofluorescence of FOXL2 (green) co-stained with DDX4 (purple) and Hoechst (blue) in day 17 ovaries in vitro with DMSO and quercetin. Scale bar, 100 μm (left); 20 μm (right). (H) Quantitative analysis of the relative follicle count in ovaries cultured with DMSO or quercetin on day 17, as shown in Figure 3G. Data are presented as the mean ± SD (n = 2 biologically independent samples); unpaired two-sided Student’s t test; NS, not significant; *P< 0.05, **P < 0.01.

To explore the potential of antioxidants to improve cell viability in cultured ovaries, a total of 10 widely utilized and orally administered antioxidants (quercetin [ 47– 49] , glutathione [50], vitamin C [ 51, 52] , ginsenoside Rg1 [ 53, 54] , curcumin [55], melatonin [ 23, 24] , coenzyme Q10 [ 56, 57] , acetylcysteine [58], metformin [ 59, 60] , and resveratrol [ 61, 62] ) were selected to evaluate their potential to facilitate the development of cultured ovaries ( Figure 3A). Through a thorough literature review, we identified the optimal concentration of each antioxidant for ovarian cell applications. On the basis of these references, we selected three experimentally tested concentrations ( Supplementary Figure S2A). Since the size of the primordial follicle pool was highly correlated with the area of ovarian tissue ( Supplementary Figure S1F), we systematically quantified the area of ovarian tissue across these concentrations and used this metric as a key criterion to determine the most effective antioxidant concentration for *in vitro* ovarian culture systems ( Figure 3B and Supplementary Figure S2B,C). Moreover, we found that the decrease in ROS levels in the quercetin group was more prominent than that in the other groups ( Figure 3C). In particular, the area of cultured ovaries significantly expanded in the quercetin group, as did the glutathione and vitamin C groups ( Figure 3D).

Next, to investigate the combined effects of quercetin coupled with glutathione or vitamin C treatment, the cultured ovaries were randomly allocated into four groups: the control and quercetin groups, as well as the quercetin + glutathione and quercetin + vitamin C combined groups ( Supplementary Figure S2D). Although the combined treatment also reduced the level of ROS and increased the area of the ovaries, the effect was not significant ( Supplementary Figure S2E,F).

Notably, compared with the control group, quercetin treatment led to an increase in the number of BVSC ^+^ cells in the cultured ovaries ( Figure 3E,F). Immunofluorescence staining also revealed a notable increase in the total follicle count in the cultured ovaries treated with quercetin, especially in the primordial follicles ( Figure 3G,H). Above all, quercetin exhibited the optimal effect in terms of reducing ROS and increasing the follicular reserve.

### Quercetin enhances the development of cultured ovaries by protecting mitochondria and reducing ROS

**Figure 4:**
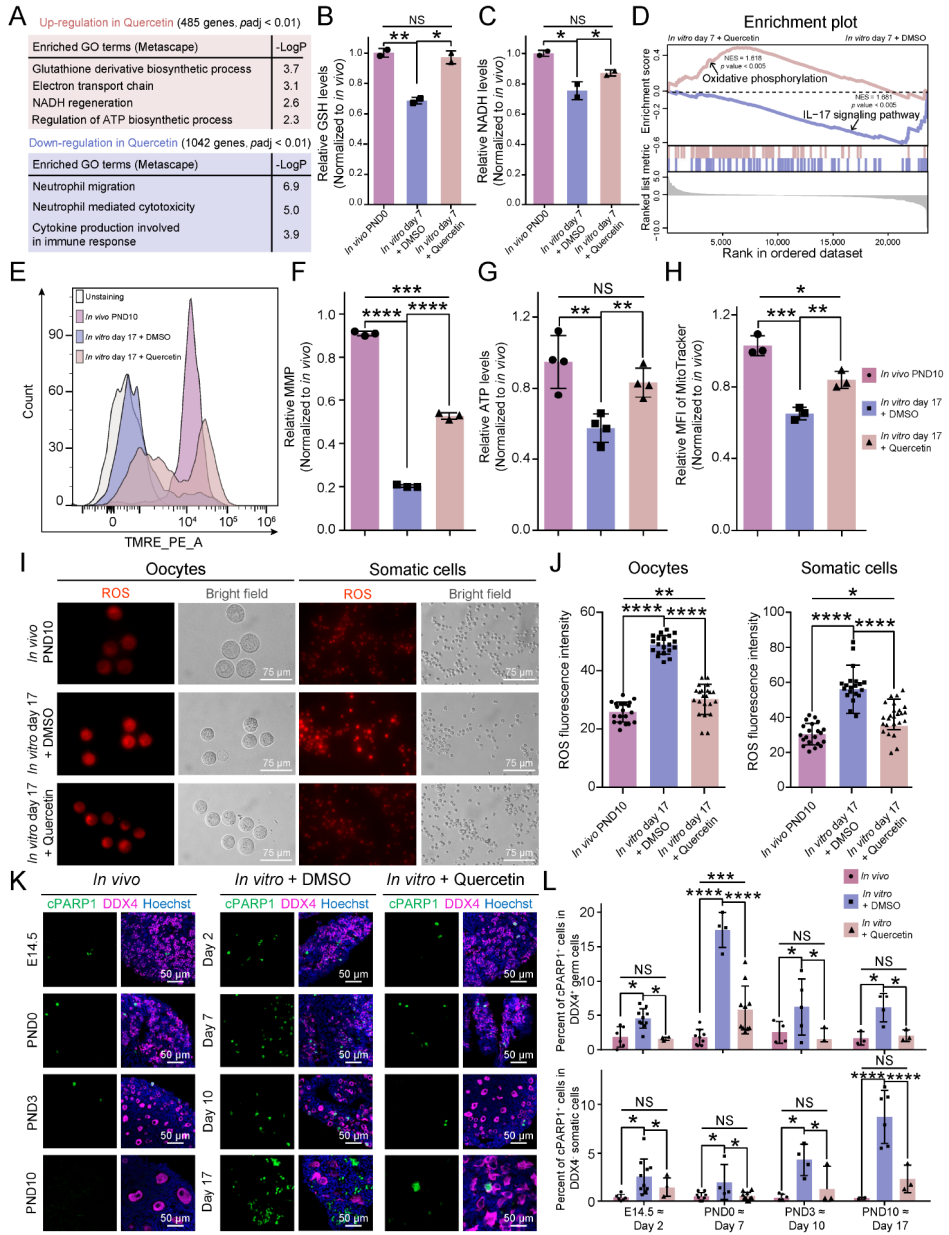
Figure 4 Quercetin rescues mitochondrial function and decreased ROS in cultured ovaries (A) Enriched GO terms (performed via the Metascape tool with a well-adopted hypergeometric test and Benjamini-Hochberg p value) of the upregulated (up) and downregulated (down) genes with quercetin treatment. (B) Quantitative analysis of relative GSH levels in PND0 in vivo and day 7 in vitro in ovaries treated with DMSO or quercetin. Data are presented as the mean ± SD (n = 2 biologically independent samples); one-way ANOVA; NS, not significant; *P < 0.05, **P < 0.01. (C) Quantitative analysis of relative NADH levels in PND0 in vivo and day 7 in vitro in ovaries treated with DMSO or quercetin. Data are presented as the mean ± SD (n = 2 biologically independent samples); one-way ANOVA; NS, not significant; *P < 0.05. (D) GSEA showing the upregulated and downregulated pathway activity in response to quercetin treatment. (E) Flow cytometry analysis of the intracellular mitochondrial membrane potential (MMP) of PND10 in vivo and day 17 in vitro in ovaries treated with DMSO or quercetin by staining with tetramethylrhodamine ethyl ester perchlorate (TMRE). (F) Quantitative analysis of relative MMP levels in PND10 in vivo and day 17 in vitro in ovaries treated with DMSO or quercetin shown in Figure 4E. Data are presented as the mean ± SD (n = 3 biologically independent samples); one-way ANOVA; ***P < 0.001, ****P < 0.0001. (G) Quantitative analysis of relative ATP levels in PND10 in vivo and day 17 in vitro in ovaries treated with DMSO or quercetin. Data are presented as the mean ± SD (n = 4 biologically independent samples); one-way ANOVA; NS, not significant; **P < 0.01. (H) Quantitative analysis of the relative mean fluorescence intensity (MFI) of MitoTracker in PND10 in vivo and day 17 in vitro in ovaries treated with DMSO or quercetin, as shown in Supplementary Figure S3F. Data are presented as the mean ± SD (n = 3 biologically independent samples); one-way ANOVA; *P < 0.05, **P < 0.01, ***P < 0.001. (I) Representative images of ROS fluorescence (red) and bright field (gray) images of oocytes (left) and somatic cells (right) at PND10 in vivo and day 17 in vitro in ovaries treated with DMSO or quercetin. The cells were imaged under an inverted fluorescence microscope using consistent parameters. Scale bar, 75 μm. (J) Quantitative analysis of the ROS fluorescence intensity of oocytes (left) and somatic cells (right) shown in Figure 4I. Data are presented as the mean ± SD with data points (20, 20 and 22 oocytes and 21, 21 and 25 somatic cells, respectively; n = 2 biologically independent samples); one-way ANOVA; *P < 0.05, **P < 0.01, ****P < 0.0001. (K) Immunostaining of cPARP1 (green) co-stained with DDX4 (purple) and Hoechst (blue) in ovaries both in vivo and in vitro after DMSO and quercetin treatment at the indicated time points. Scale bar, 50 μm. (L) Quantitative analysis of the percentages of cPARP1+ cells in DDX4+ germ cells (top) and DDX4- somatic cells (bottom) in Figure 4K. Data are represented as the mean ± SD (n ≥ 2 biologically independent samples); one-way ANOVA; NS, not significant; *P < 0.05; ****P < 0.0001.

To further elucidate the mechanisms of quercetin in cultured ovaries, we performed transcriptome analysis via RNA sequencing ( Supplementary Figure S3A). Following the quality control procedures, a series of differentially expressed genes (DEGs) were identified ( Supplementary Figure S3B,C). GO enrichment analysis demonstrated that quercetin could downregulate pathways associated with cytotoxicity, such as “neutrophil mediated cytotoxicity” and “cytokine production involved in immune response” ( Figure 4A). Interestingly, quercetin treatment upregulated the expression of genes involved in the glutathione (GSH) and NADH biosynthesis pathways ( Figure 4A). Further analysis showed that, compared with those in *in vivo* conditions, the intracellular levels of both GSH and NADH were lower *in vitro*; notably, quercetin treatment effectively reversed these reductions in ovaries cultured *in vitro* ( Figure 4B,C). Moreover, GSEA revealed significant upregulation of the oxidative phosphorylation (OXPHOS) gene set in response to quercetin treatment, which was crucial for adenosine triphosphate (ATP) generation in mitochondria and the maintenance of the intracellular redox state ( Figure 4D). Moreover, the IL-17 signaling pathway was markedly altered upon quercetin treatment ( Figure 4D), which is consistent with the previously observed suppression of the inflammatory response by quercetin. The qPCR results verified the expression patterns of marker genes within the aforementioned pathways ( Supplementary Figure S3D,E).

Notably, quercetin treatment upregulated the expression of genes associated with mitochondria-related GO terms, including those involved in the “regulation of ATP biosynthetic process” ( Figure 4A). Further experiments revealed that quercetin rescued the waning mitochondrial membrane potential (MMP) and ATP levels in cultured ovaries ( Figure 4E–G). In particular, the ATP level was very close to that *in vivo*. In addition, quercetin treatment enhanced mitochondrial characteristics, with a greater number of mitochondria and a more uniform distribution, as detected via MitoTracker Red FM ( Figure 4H and Supplementary Figure S3F,G). Moreover, quercetin treatment diminished the intracellular ROS levels in both oocytes and somatic cells at both the early stage (day 7) and the late stage (day 17) ( Figure 4I,J and Supplementary Figure S3H,I). Finally, we observed a marked decrease in the activation of cPARP1 in both oocytes and somatic cells during the entire culture process, including the early stages, with the addition of quercetin ( Figure 4K,L). Notably, the levels of cell apoptosis at many stages approximated those observed under *in vivo* conditions, such as in oocytes on day 10 and in somatic cells on day 7. These findings were directly associated with our findings that quercetin treatment increased follicle yield ( Figure 3D–H). Collectively, these results indicated that quercetin promoted the development of cultured ovaries by safeguarding mitochondrial function and attenuating ROS accumulation.

### Applications of ovarian explant culture in modelling fetal reproductive aberrations of GDM-affected F1 female offspring

Primordial follicles are established during the embryonic period, making the development of the female reproductive system highly susceptible to the intrauterine environment of the mother [ 63, 64] . It has been reported that as early as the fetal stage, maternal gestational diabetes mellitus (GDM) severely affects the development of the reproductive system in F1 female offspring [ 65– 68] . Moreover, considering the inaccessibility and ethical restrictions of human fetal ovary tissue, as well as the limitation of traditional cell cultures in accurately mimicking ovarian disease pathology, it is essential to establish a drug screening platform based on this mouse ovarian explant 3D culture model and to help alleviate ovarian alterations in GDM-affected female offspring.

**Figure 5:**
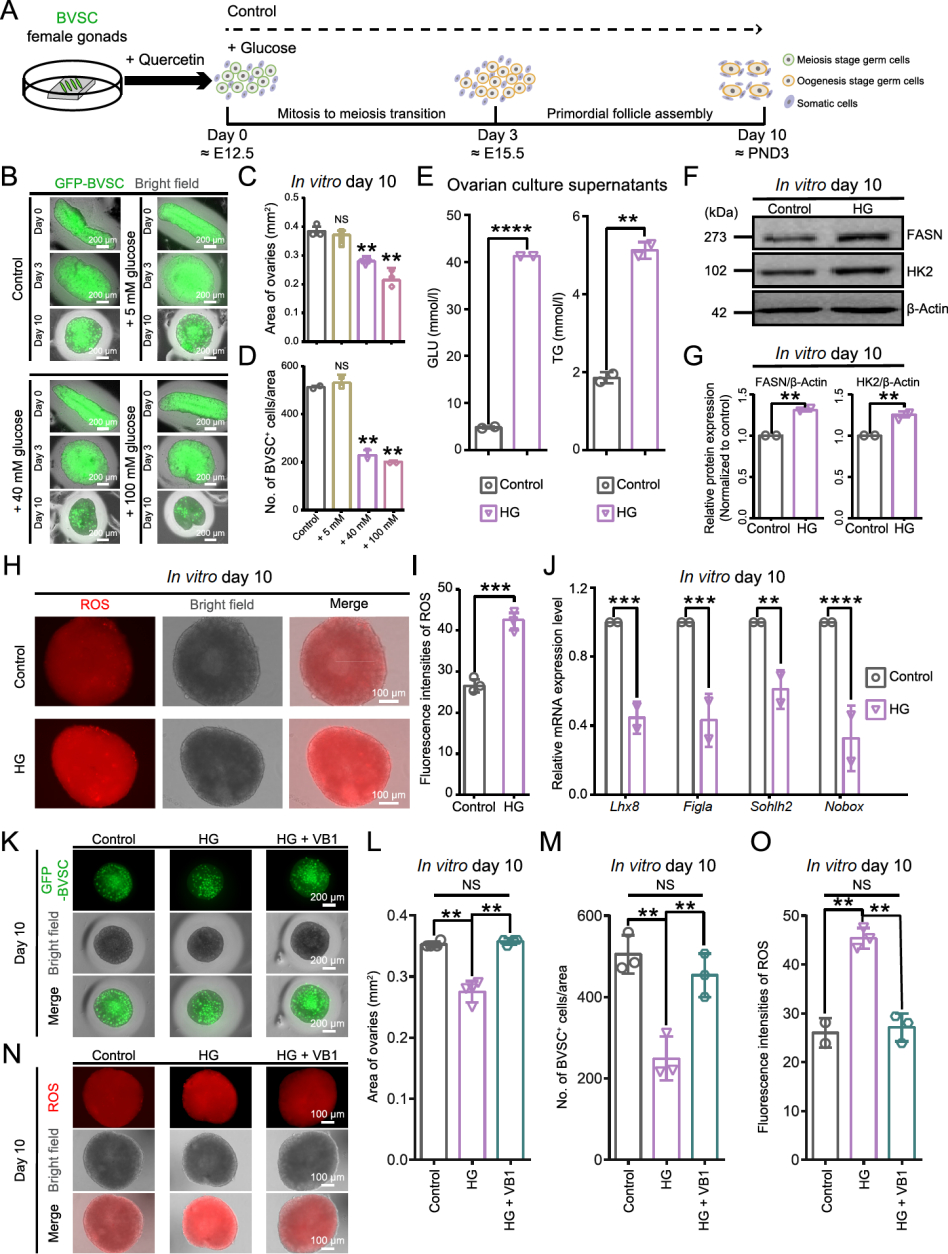
Figure 5 An ovarian model of GDM-affected F1 female offspring is established (A) Schematic illustration of ovaries in vitro under control culture conditions and different glucose concentration groups. (B) Representative images of GFP-BVSC fluorescence (green) and bright field (gray) images of ovaries in vitro at the indicated time points in each group. Scale bar, 200 μm. (C) Quantitative analysis of the ovarian area in the ovaries cultured on day 10 in each group, corresponding to Figure 5B. The data are presented as the mean ± SD (n = 2 biologically independent samples); one-way ANOVA; NS, not significant; **P < 0.01. (D) Quantitative analysis of the number of BVSC+ cells in the ovaries cultured on day 10 in each group, corresponding to Figure 5B. Data are presented as the mean ± SD (n = 2 biologically independent samples); one-way ANOVA; NS, not significant; **P < 0.01. (E) Box plots showing the levels of glucose (GLU) and triglycerides (TG) in ovarian culture supernatants from control and high glucose (HG) culture conditions; unpaired two-sided Student’s t test; **P < 0.01, ****P < 0.0001. (F) Immunoblots of the indicated proteins in ovaries cultured on day 10 under control and HG culture conditions. (G) The relative expression levels of the proteins in Figure 5F; unpaired two-sided Student’s t test; **P < 0.01. (H) Representative images of ROS fluorescence (red) and bright field (gray) images of ovaries cultured on day 10 under control and high glucose (HG) culture conditions. The cells were imaged under an inverted fluorescence microscope using consistent parameters. Scale bar, 100 μm. (I) Quantitative analysis of the ROS fluorescence intensity shown in Figure 5H. Data are presented as the mean ± SD (n = 2 biologically independent samples); unpaired two-sided Student’s t test; ***P < 0.001. (J) qPCR analysis of the expression of marker genes in ovaries cultured on day 10 under control and HG culture conditions. Data are presented as the mean ± SD (n = 2 biologically independent samples); unpaired two-sided Student’s t test; **P < 0.01, ***P < 0.001, ****P < 0.0001. (K) Representative images of GFP-BVSC fluorescence (green) and bright field images (gray) of ovaries cultured on day 10. Scale bar, 200 μm. (L) Quantitative analysis of the ovarian area in the ovaries cultured on day 10, corresponding to Figure 5K. Data are presented as the mean ± SD (n = 2 biologically independent samples); one-way ANOVA; NS, not significant; **P < 0.01. (M) Quantitative analysis of the number of BVSC+ cells in the ovaries cultured on day 10, corresponding to Figure 5K. Data are presented as the mean ± SD (n = 2 biologically independent samples); one-way ANOVA; NS, not significant; **P < 0.01. (N) Representative images of ROS fluorescence (red) and bright field (gray) images of ovaries cultured on day 10. The cells were imaged under an inverted fluorescence microscope using consistent parameters. Scale bar, 100 μm. (O) Quantitative analysis of ROS fluorescence intensity in ovaries cultured on day 10, corresponding to Figure 5N. Data are presented as the mean ± SD (n = 2 biologically independent samples); one-way ANOVA; NS, not significant; **P < 0.01.

The exposure of cultured ovaries (treated with quercetin) to high glucose triggered a cascade of developmental perturbations, mirroring the aberrant phenotypes observed in the ovaries of GDM-affected F1 female offspring. The ovaries were randomly grouped and cultured in different glucose concentration groups: control, 5 mM, 40 mM and 100 mM glucose ( Figure 5A,B and Supplementary Figure S4A). Compared with those in the control group, the ovaries in the 5 mM glucose group were not significantly different. In contrast, ovaries cultured in 40 mM glucose presented a significant decrease in both the ovarian area and the number of BVSC ^+^ oocytes on day 10, which was equivalent to PND3 (the key time point for the formation of the primordial follicle pool) ( Figure 5C,D). This was accompanied by a decrease in the expression of meiosis-related genes and an increase in the expression of inflammation- and apoptosis-related genes on day 3, equivalent to E15.5, the period of mitosis to meiosis transition ( Supplementary Figure S4B,C). The overall relatively severe phenotypes at the higher concentration of 100 mM were similar to those observed at 40 mM; therefore, 40 mM glucose was used for modelling the high glucose (HG) state.

We subsequently assessed GDM-specific molecular markers in cultured ovaries exposed to HG and revealed that HG treatment increased the levels of triglycerides, which was consistent with the trends observed in GDM patients [66] ( Figure 5E). Additionally, HG treatment increased the expression of enzymes involved in glucose metabolism, such as FASN (a key enzyme in triglyceride synthesis) and HK2 (a key enzyme involved in glycolysis) ( Figure 5F,G). In line with the insulin resistance characteristic of GDM, HG treatment downregulated the expression of genes associated with glucose tolerance and glucose transport [ 69, 70] ( Supplementary Figure S4D). Together, these results suggested that our ovarian model with HG stimulation could serve as a useful model for studying ovarian diseases in GDM-affected F1 female offspring.

An enhanced oxidative stress status has been documented in diabetic animals as well as in fetal ovaries affected by maternal obesity [ 66, 71, 72] . In detail, HG treatment also elicited much stronger ROS signals in the cultured ovaries ( Figure 5H,I and Supplementary Figure S4E,F). Furthermore, the expression of oocyte-specific genes ( *Lhx8*, *Figla*, *Sohlh2*, and *Nobox*), which are crucial for primordial follicle formation, was downregulated in the HG group ( Figure 5J). Additionally, HG treatment led to an increase in cell death ( Supplementary Figure S4G,H). Collectively, these results indicated that high glucose environments led to an overproduction of ROS and impaired the survival of ovarian cells, ultimately inhibiting ovarian growth, which could essentially mimic the aberrant traits observed in the ovaries of GDM-affected F1 female offspring.

Vitamin B1 has been reported to be useful in the reproductive system of GDM-affected F1 female offspring [66]. Indeed, vitamin B1 significantly increased the ovarian area and the number of BVSC ^+^ cells ( Figure 5K–M). Importantly, the ROS signal was significantly reduced upon treatment with vitamin B1 ( Figure 5N,O), which is consistent with the findings of a previous study [66]. Notably, vitamin B1 was able to rescue these GDM-like phenotypes ( Supplementary Figure S4I,J). These findings highlighted that this ovarian model with HG stimulation could erve as a valuable model for screening drugs that could protect the reproductive health of GDM-affected F1 female offspring.

## Discussion

In recent years, 3D culture methods, including the extension of 3D organoid culture, have been increasingly applied to investigate the biology and pathology of the female reproductive tract (FRT), including the ovaries, endometrium and cervix, as well as the cancer tissues of the FRT, owing to their ability to closely mimic the structure and function of the tissues from which they are derived [ 3, 38, 73, 74] . Despite these advantages, standard *in vitro* culture conditions inevitably lead to elevated levels of ROS, which are considered one of the primary factors contributing to impaired female reproductive development, particularly affecting ovarian health [ 75, 76] . In this study, we improved the ovarian explant culture system in several aspects: (1) we developed a visualized ovarian explant 3D culture model with the GFP-BVSC reporter system, enabling non-invasive monitoring of comprehensive oocyte and follicle development; (2) we discovered that quercetin, a plant-derived natural antioxidant, could improve the development of cultured ovaries, consequently facilitating the formation of a high-yield and high-quality early follicle pool; and (3) we constructed a user-friendly model for studying the female reproductive health of GDM-affected female offspring at the fetal stage. In the future, this ovarian explant 3D culture model could serve as a rapid monitoring and screening tool for ovarian disease modelling as well as drug research. Moreover, leveraging advanced microscopy techniques, this model could serve as an instrumental means for conducting real-time observations of the morphology and mechanics of ovarian cells.

*In vitro* ovarian models have greatly advanced the study of female reproduction. The primary distinction between our 3D ovarian explant model and traditional organoid models lies in the tissue origin: the 3D ovarian explant model utilized ovarian tissues derived from fetal female gonads in our study, as well as ovarian tissue fragments in previous studies [ 3, 77] . In contrast, organoids are typically generated from stem cells ( *e.g.*, pluripotent or adult stem cells) that self-organize into structured systems through proliferation and differentiation [5]. Previous studies have demonstrated the differentiation of mouse and human pluripotent stem cells into primordial germ cell-like cells (PGCLCs) and fetal ovarian somatic cell-like cells (FOSLCs) [ 6, 78] . Mouse PGCLCs (mPGCLCs) have the potential to form ovarian follicles or organoids when co-cultured with ovarian somatic cells or mouse FOSLCs (mFOSLCs), with some models even generating mature oocytes [ 6, 39] . However, the differentiation efficiency and reproducibility of traditional organoids remain challenging [79]. In this context, our 3D ovarian explant model has certain advantages: (1) mouse fetal gonadal tissues are readily obtainable, facilitating reproducibility and stability; (2) the culture system is easy to implement and can maintain structural integrity over extended culture time; and (3) the model effectively recapitulates early ovarian development and folliculogenesis within a more physiology-like microenvironment.

Oxidative stress, resulting from an imbalance between pro-oxidants and antioxidants, can lead to female infertility, which may be attributed to diminished oocyte quality or damage to ovarian somatic cells. This imbalance often arises from increased levels of pro-oxidants, such as ROS, or a decrease in antioxidant defense mechanisms [ 80, 81] . Under physiological conditions, ROS are involved in various signaling pathways that regulate ovarian development. In the present study, we observed increased levels of ROS in cultured ovaries compared with those in ovaries *in vivo*. Moreover, there was a notable increase in cell mortality among both oocytes and somatic cells, indicating the urgent need for more research to identify the optimal redox balance in cultured ovaries.

Notably, the imbalance between ROS generation and the antioxidant defense system contributes to the development of ovarian diseases [13]. Antioxidants have been widely applied in the treatment of ovarian disorders. For example, quercetin can alleviate oocyte aging by reducing oxidative stress, thereby improving oocyte quality *in vivo* [ 82, 83] . Moreover, quercetin treatment relieves deterioration in oocytes, improves subsequent embryo development, and enhances the viability of ovarian granulosa cells *in vitro* [ 49, 84, 85] . Additionally, quercetin can enhance the morphological integrity and follicular maturity of *Bos indicus* ovarian fragments in *in vitro* culture [86]. Vitamin C can protect against cisplatin-related infertility by suppressing oxidative stress and apoptosis in the ovaries [87]. Glutathione can alleviate cadmium exposure-induced meiotic defects in porcine oocytes by eliminating excessive ROS [88]. On the basis of these lines of evidence, incorporating antioxidant treatment into our ovarian culture system could improve oocyte quality and overall ovarian function, but further in-depth research is warranted in the future.

A wealth of epidemiological and experimental data support the notion that exposure to adverse intrauterine conditions in early life can significantly increase the risk of developing diseases later in life [ 89, 90] . GDM is one of the most common conditions associated with the “fetal origins of adult disease” [91]. The development and function of the ovary are highly susceptible to the maternal intrauterine environment because the primordial follicle pool is formed during the embryonic phase [64]. Many studies have indicated that GDM adversely affects the ovarian function of female offspring as early as the embryonic stage [ 65, 66, 68, 92] . Moreover, since the approach to ensuring the reproductive health of GDM-affected F1 female offspring remains elusive, cultured ovaries mimicking the development of the fetal ovary are useful for studying the underlying mechanisms and therapeutic strategies. Thus, on the basis of this visualized and optimized ovarian explant culture platform, we constructed a user-friendly model for studying the reproductive health of GDM-affected female offspring. Our future work will focus on conducting drug screening and exploring treatment regimens on the basis of this model.

In summary, we propose a visualized ovarian explant 3D culture system in which quercetin promotes the spatial and temporal growth of ovaries by improving the yield and quality of the initial follicle pool. Importantly, this ovarian explant 3D culture model can serve as a valuable tool for ovarian disease modelling and drug screening, such as in GDM-affected F1 ovarian models. In conclusion, an accessible and convenient ovarian explant culture platform is established, facilitating ovarian disease research and potential treatment exploration.

## Supporting information

25118Supplementary_data-20250519

## Supplementary Data

Supplementary data is available at *Acta Biochimica et Biphysica Sinica* online.
