# Supplementary material for: A visualized and quercetin-optimized three-dimensional culture model of mouse ovaries derived from fetal gonads: A visualized and quercetin-optimized mouse ovarian 3D culture model
Source: Acta Biochim Biophys Sin (Shanghai). 2025 Jun 3;58(2):275–89. doi: 10.3724/abbs.2025084 (PMC12900695; doi:10.3724/abbs.2025084)
Supplement: 25118Supplementary_data-20250519 [file 25118Supplementary_data-20250519.docx]

**
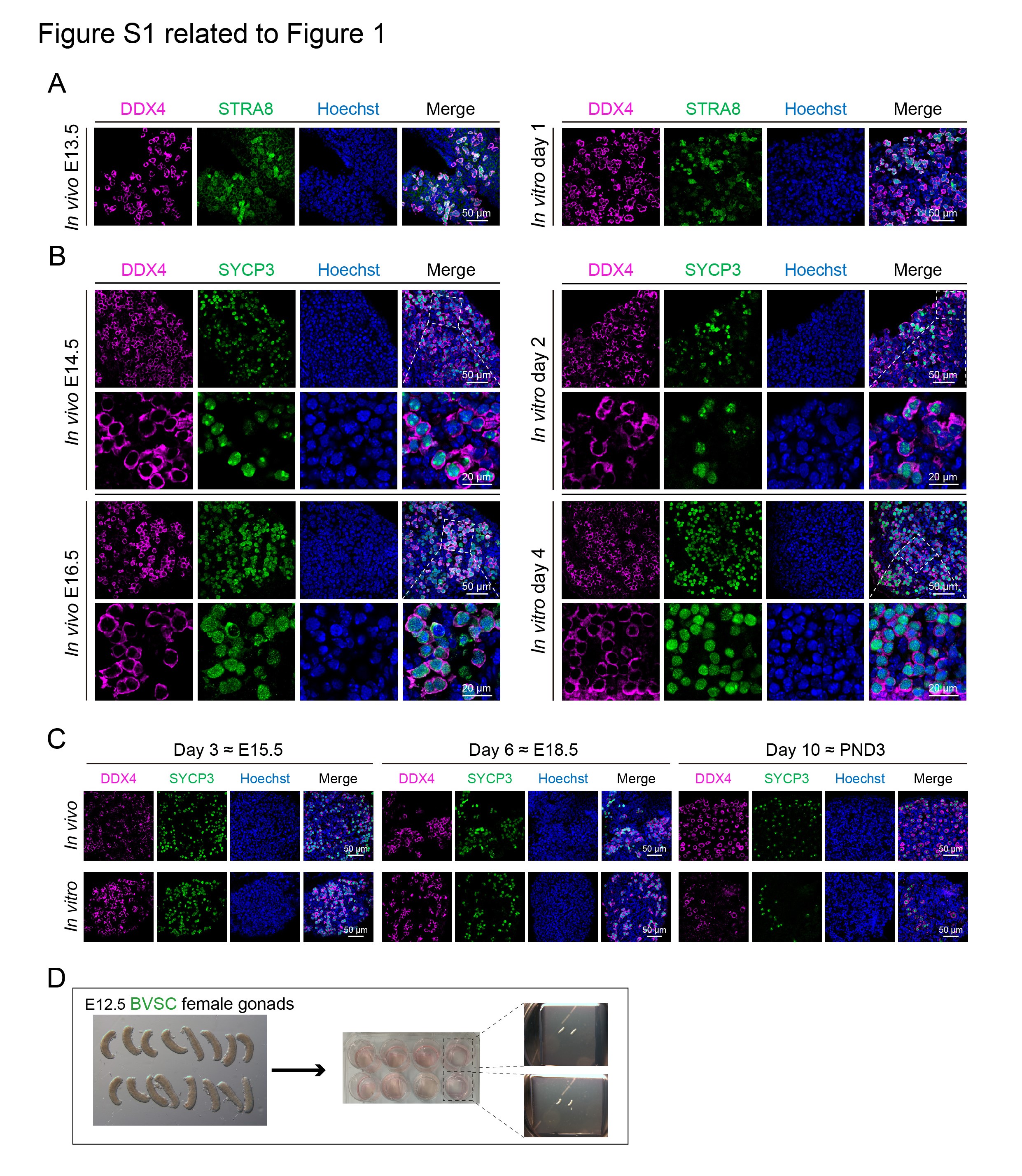
**

**
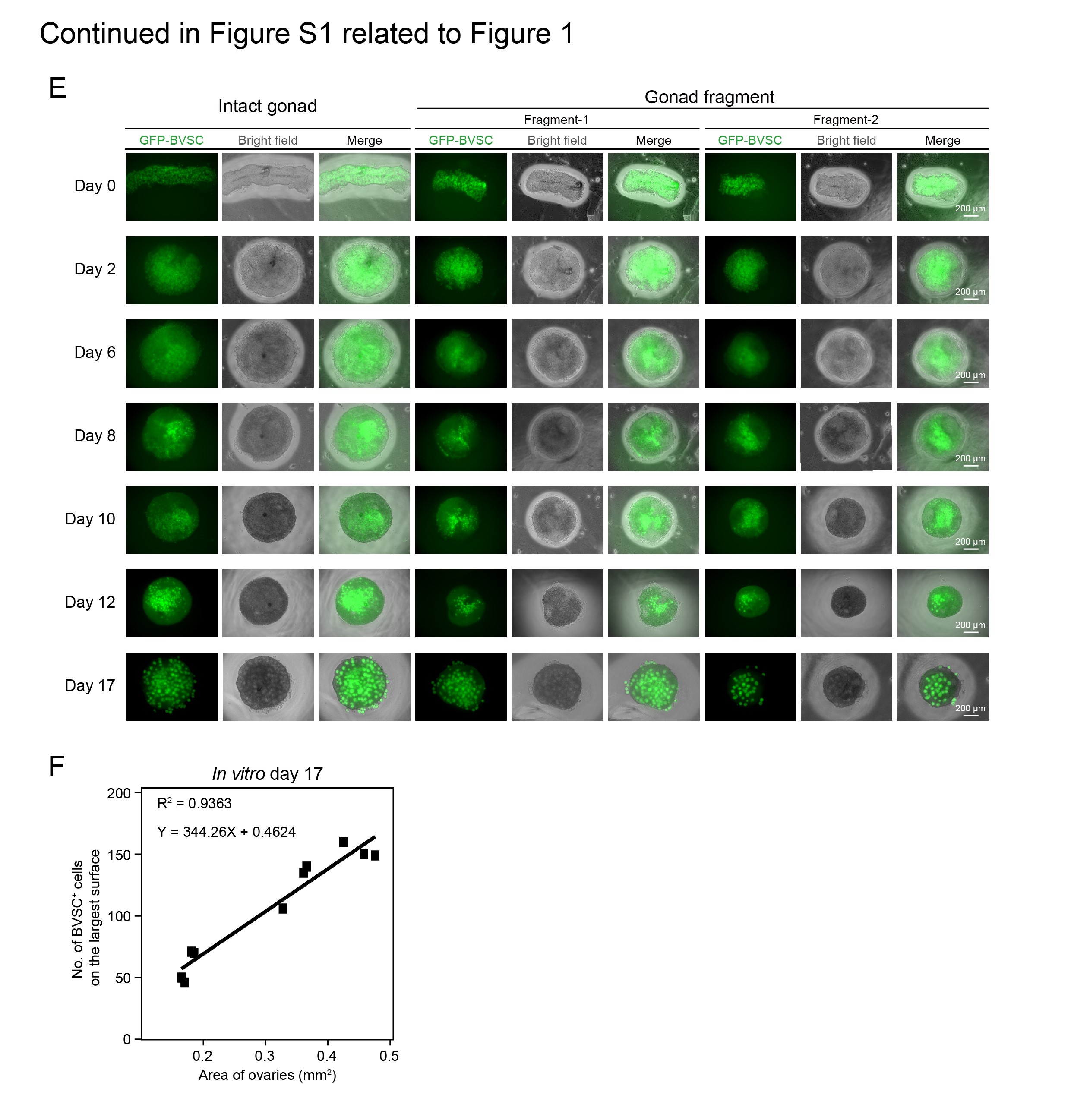
**

**Supplementary Figure S1. *In vitro* reconstitution of meiosis in organ-cultured ovaries** (A) Immunostaining of STRA8 (green) co-stained with DDX4 (purple) and Hoechst (blue) in ovaries both *in* *vivo* and *in* *vitro*. Scale bar, 50 μm. (B,C) Immunostaining of SYCP3 (green) co-stained with DDX4 (purple) and Hoechst (blue) in ovaries both *in* *vivo* and *in* *vitro*. Scale bar, 50 μm (up) and 20 μm (down). (D) Schematic diagram of collecting female gonads for *in vitro* culture. (E) Representative images of GFP-BVSC fluorescence (green) and bright field (gray) of the cultured ovaries (intact gonad (left) and gonad fragment (right)) at the indicated time points. Scale bar, 200 μm. (F) A scatter plot showing the quantification of BVSC⁺cell numbers per mm^2^ of ovarian tissue cultured on day 17, with linear regression analysis performed using GraphPad Prism (R² = 0.9363).


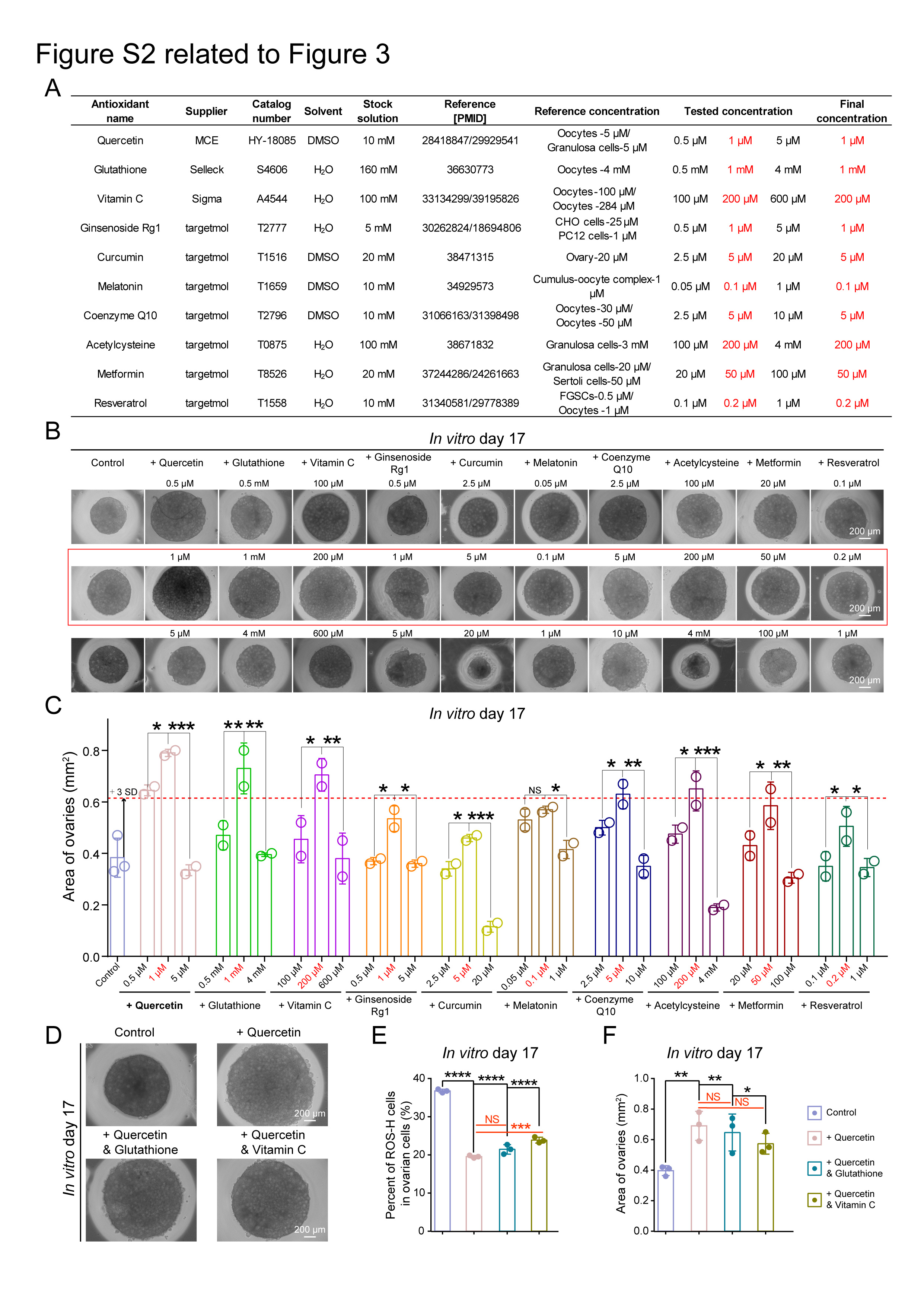


**Supplementary Figure S2. Selection of antioxidants to enhance the growth efficiency of cultured ovaries**  (A) The trilinear table showing antioxidant data, including reference concentrations and tested concentrations. CHO cells, Chinese hamster ovary cells; PC12 cells, pheochromocytoma cells; FGSCs, female germline stem cells. (B) Bright field images of cultured ovaries in various culture media on day 17. Scale bar, 200 μm. (C) Quantitative analysis of the ovarian area in various culture media on day 17. Red dotted lines indicate the + 3 SD of control group. Data are presented as the mean ± SD (*n*  ≥ 2 biologically independent samples); one-way ANOVA; NS is not significant, **P* < 0.05, ***P* < 0.01, and ****P* < 0.001. (D) Bright field images of the cultured ovaries in various culture media at the indicated time points. Scale bar, 200 μm. (E) Quantitative analysis of ROS-H cells in various culture media in the ovaries cultured on day 17, corresponding to **Figure S2D**. Data are presented as the mean ± SD (*n* = 3 biologically independent samples); one-way ANOVA; NS is not significant, ****P <* 0.001, *****P <* 0.0001. (F) Quantitative analysis of the ovarian area in various culture media in the ovaries cultured on day 17, corresponding to **Figure S2D**. Data are presented as the mean ± SD (n ≥ 2 biologically independent samples); one-way ANOVA; NS is not significant, **P <* 0.05, ***P <* 0.01.


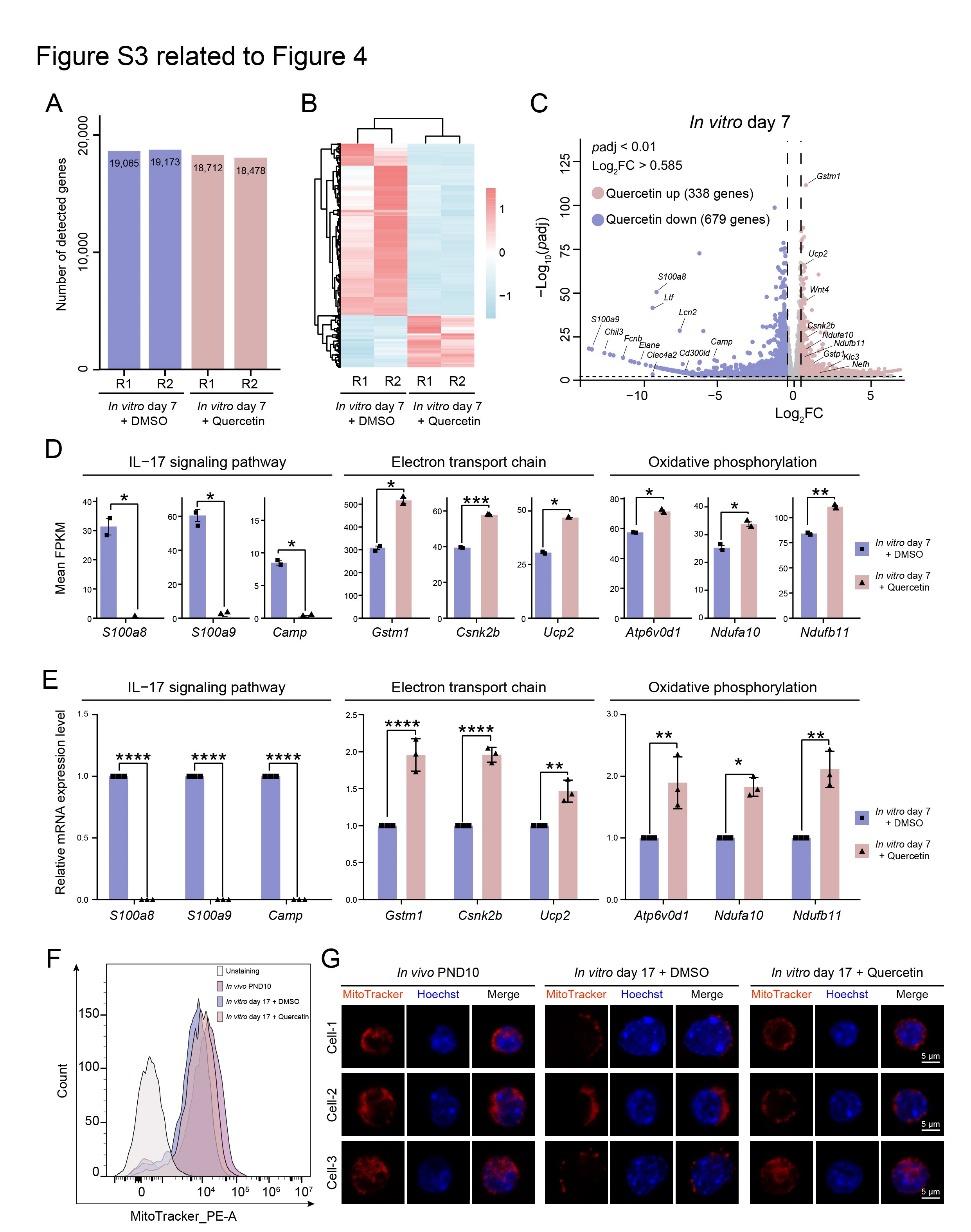


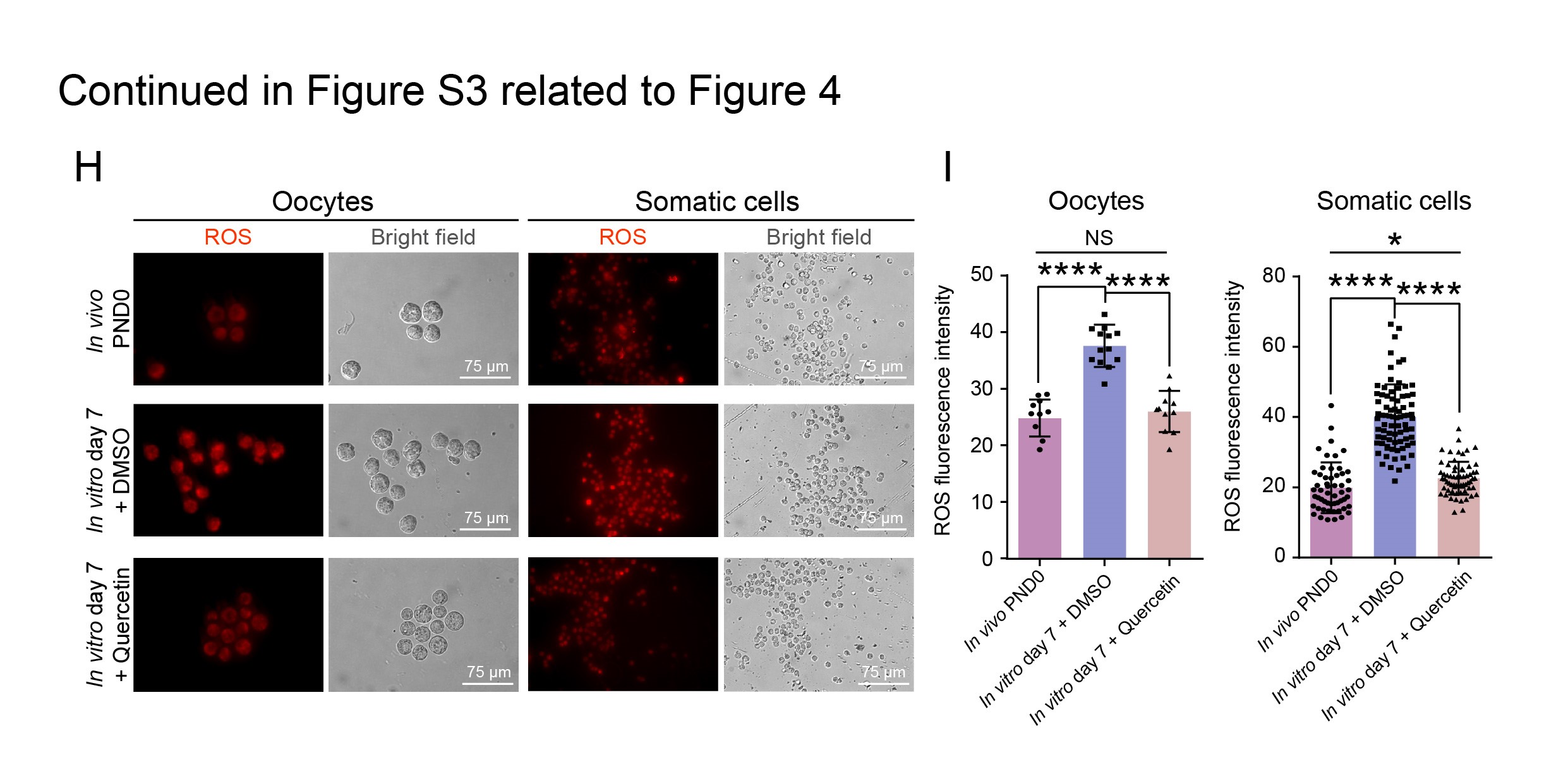


**Supplementary Figure S3. Quercetin restores mitochondrial function and attenuates ROS accumulation in cultured ovaries**  (A) Bar plot showing the number of detected genes after quality control. (B) Heatmap showing the expression of DEGs (*p*adj < 0.01). (C) Volcano plot showing the upregulated (red dot) and downregulated (blue dot) genes with quercetin treatment. (D) Bar chart showing the expression levels (mean FPKM) of represented DEGs; unpaired two-sided Student’s *t*-test; **P <* 0.05, ***P <* 0.01, ****P <* 0.001. (E) qPCR analyzing the expression levels of represented DEGs in **Figure S3D**. Data are presented as the mean ± SD (n = 2 biologically independent samples); unpaired two-sided Student’s *t*-test; **P <* 0.05, ***P <* 0.01, *****P <* 0.0001. (F) Flow cytometry analysis of intracellular mitochondria in PND10 *in vivo* and day 17 *in vitro* ovaries treated with DMSO and Quercetin, stained with 50 nM MitoTracker Red FM. (G) Cell staining results of intracellular mitochondria in PND10 *in vivo* and day 17 *in vitro* ovaries treated with DMSO and Quercetin, stained with 150 nM MitoTracker Red FM. (H) Representative images of ROS fluorescence (red) and bright field (gray) of oocytes (left) and somatic cells (right) in PND0 *in vivo* and day 7 *in vitro* ovaries with DMSO and Quercetin treatment. Cells were imaged under an inverted fluorescence microscope using consistent parameters. Scale bar, 75 μm. (I) Quantitative analysis of ROS fluorescence intensity of oocytes (left) and somatic cells (right) in **Figure S3H**. Data are presented as the mean ± SD with data points (10, 13 and 11 oocytes, 54, 78 and 64 somatic cells, respectively; *n* = 2 biologically independent samples); one-way ANOVA; NS is not significant, **P* < 0.05, *****P <* 0.0001.


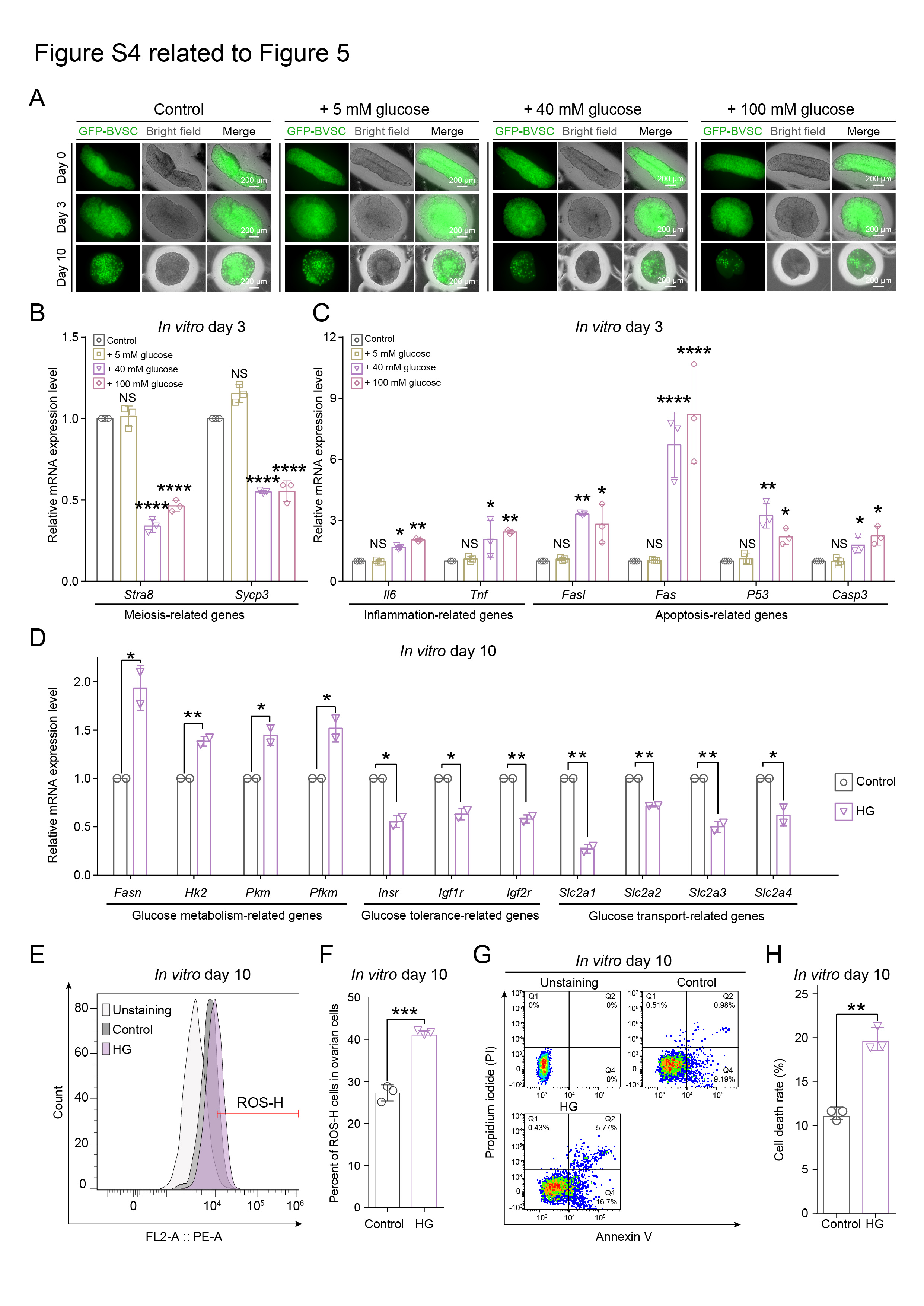


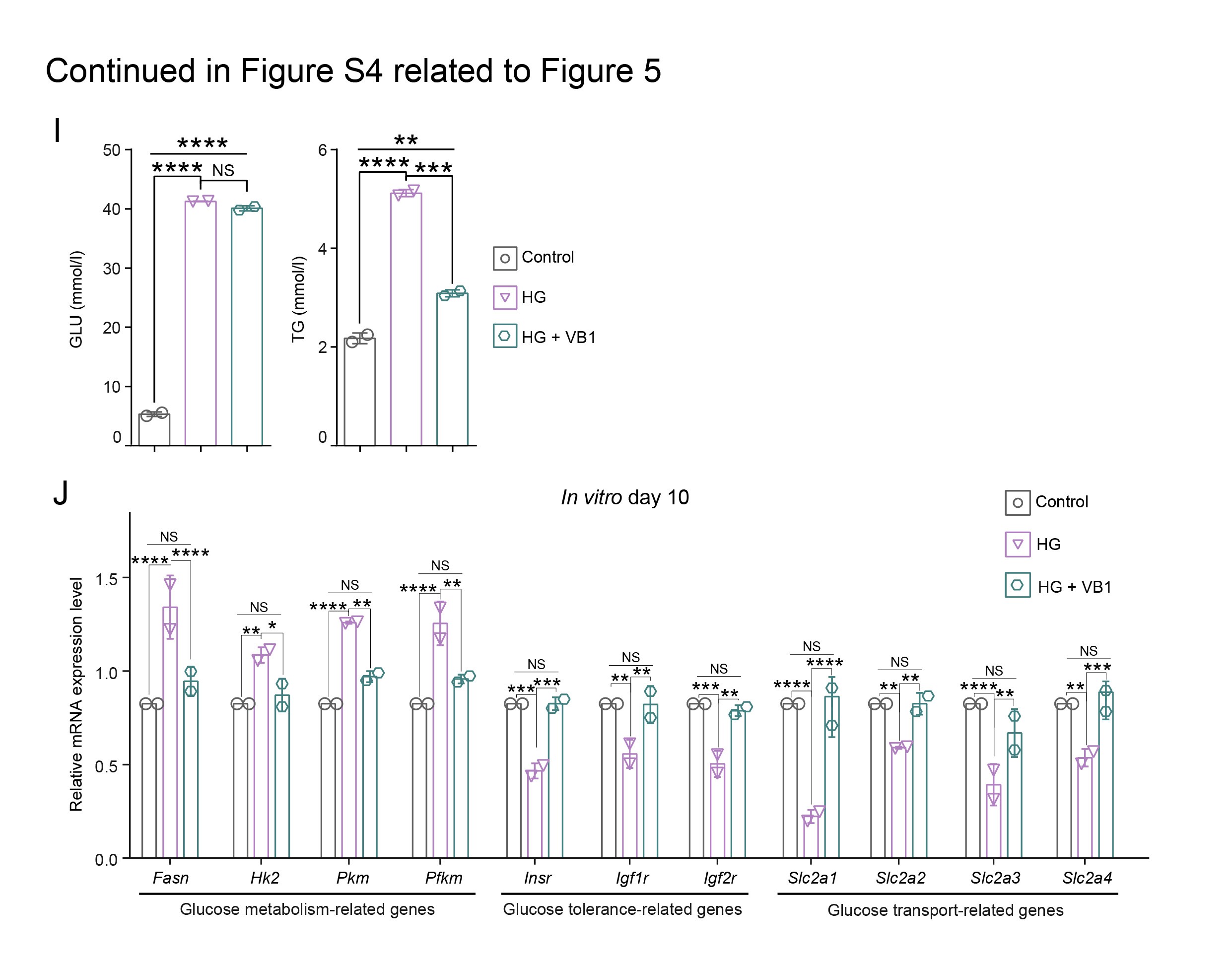


**Supplementary Figure S4. Characterization of aberrant ovarian phenotypes in cultured ovaries exposed to high glucose**  (A) Representative images of GFP-BVSC fluorescence (green) and bright field (gray) of ovaries *in vitro* at the indicated time points in each group. Scale bar, 200 μm. (B) qPCR analyzing the expressions of *Stra8* and *Sycp3* in the ovaries cultured on day 3 in each group. Data are presented as the mean ± SD (*n* = 2 biologically independent samples); one-way ANOVA; NS is not significant, *****P <* 0.0001. (C) qPCR analyzing the expressions of marker genes in the ovaries cultured on day 3 in each group. Data are presented as the mean ± SD (*n* = 2 biologically independent samples); one-way ANOVA; NS is not significant, **P <* 0.05, ***P <* 0.01, *****P <* 0.0001. (D) qPCR analyzing the expressions of marker genes in the ovaries cultured on day 10 with control and high glucose (HG) culture conditions. Data are presented as the mean ± SD (*n* = 2 biologically independent samples); unpaired two-sided Student’s *t*-test; **P* < 0.05, ***P* < 0.01. (E) Flow cytometry analysis of intracellular ROS in the ovaries cultured on day 10 with control and high glucose (HG) culture conditions through staining with dihydroethidium. PE-A^+^ cells, the indicator of ROS-H cells. (F) Quantitative analysis of ROS-H cells in **Figure S4E**. Data are presented as the mean ± SD (*n* = 3 biologically independent samples); unpaired two-sided Student’s *t*-test; ****P <* 0.001. (G) Flow cytometry analysis of cell apoptosis in the ovaries cultured on day 10 with control and high glucose (HG) culture conditions through double staining with annexin V and PI. Annexin V^+^ PI^−^ cells, the indicator of early-stage apoptotic cells; Annexin V^+^ PI^+^ cells, the indicator of late-stage apoptotic cells and other kinds of dead cells. (H) Quantitative analysis of cell death rate in **Figure S4G**. Data are presented as the mean ± SD (*n*= 2 biologically independent samples); unpaired two-sided Student’s *t*-test; ***P <* 0.01. (I) Box plots showing the levels of glucose (GLU) and triglycerides (TG) in ovarian culture supernatants; one-way ANOVA; NS is not significant, ***P* < 0.01, ****P* < 0.001,*****P* < 0.0001. (J) qPCR analyzing the expressions of marker genes in the ovaries cultured on day 10. Data are presented as the mean ± SD (n = 2 biologically independent samples); one-way ANOVA; **P* < 0.05, ***P* < 0.01, ****P* < 0.001, *****P* < 0.0001.

| **Supplementary Table S1. The sequences of primers used in this study** | | |
| --- | --- | --- |
|  | **Gene name** | **Primer sequence (5**′→**3**′**)** |
| Figure 2H | *Tnf*-q-F | GCCTCTTCTCATTCCTGCTT |
|  | *Tnf*-q-R | CTCCTCCACTTGGTGGTTTG |
|  | *Il6*-q-F | GTTCTCTGGGAAATCGTGGA |
|  | *Il6*-q-R | GGTACTCCAGAAGACCAGAGGA |
|  | *Bax*-q-F | TGAAGACAGGGGCCTTTTTG |
|  | *Bax*-q-R | AATTCGCCGGAGACACTCG |
|  | *Bcl2*-q-F | AGCGTCAACAGGGAGATGTC |
|  | *Bcl2*-q-R | AGCAGGGTCTTCAGAGACAG |
|  | *Casp3*-q-F | TGGTGATGAAGGGGTCATTTATG |
|  | *Casp3*-q-R | TTCGGCTTTCCAGTCAGACTC |
|  |  |  |
| Figure 5J | *Lhx8*-q-F | CAGTTCGCTCAGGACAACAA |
|  | *Lhx8*-q-R | CCTGCAGTTCTGAAACCACA |
|  | *Figla*-q-F | CCGCCATCTGTAGGCTCAAG |
|  | *Figla*-q-R | ACACAGCCGAGTATCTGTATGTA |
|  | *Sohlh2*-q-F | TCTCAGCCACATCACAGAGG |
|  | *Sohlh2*-q-R | GGGGACGCGAGTCTTATACA |
|  | *Nobox*-q-F | CTATCCTGACAGTGACAAACGCC |
|  | *Nobox*-q-R | CACCCTCTCAGCACCCTCATTAT |
|  |  |  |
| Figure S3E | *S100a8*-q-F | AAATCACCATGCCCTCTACAAG |
|  | *S100a8*-q-R | CCCACTTTTATCACCATCGCAA |
|  | *S100a9*-q-F | GCACAGTTGGCAACCTTTATG |
|  | *S100a9*-q-R | TGATTGTCCTGGTTTGTGTCC |
|  | *Camp*-q-F | CAGCAGTCCCTAGACACCAAT |
|  | *Camp*-q-R | ACAGTCTCCTTCACTCGGAACC |
|  | *Gstm1*-q-F | GGGCCTGGACTTTCCCAATC |
|  | *Gstm1*-q-R | AATGTCTGCACGGATCCTCTCC |
|  | *Csnk2b*-q-F | AATGAGCAGGTGCCTCACTAT |
|  | *Csnk2b*-q-R | TGTTCGATCAAGTCGCTCTGG |
|  | *Ucp2*-q-F | CAGCGCCAGATGAGCTTTG |
|  | *Ucp2*-q-R | GGAAGCGGACCTTTACCACA |
|  | *Atp6v0d1*-q-F | GCTACTTGGAGGGATTAGTGCG |
|  | *Atp6v0d1*-q-R | GCGGAACTCTACTACCATCTTCT |
|  | *Ndufa10*-q-F | ACCTTTCACTACCTGCGGATG |
|  | *Ndufa10*-q-R | GTACCCAGGGGCATACTTGC |
|  | *Ndufb11*-q-F | CCTCCAGGGCTGTAATCGC |
|  | *Ndufb11*-q-R | GGTTCTTCGCGTAGACGTTTTC |
|  |  |  |
| Figure S4B | *Stra8*-q-F | GCCGGACCTCATGGAATTTGA |
|  | *Stra8*-q-R | TCACTTCATGTGCAGAGATGATG |
|  | *Sycp3*-q-F | AGCCAGTAACCAGAAAATTGAGC |
|  | *Sycp3*-q-R | CCACTGCTGCAACACATTCATA |
|  |  |  |
| Figure S4C | *Il6*-q-F | GTTCTCTGGGAAATCGTGGA |
|  | *Il6*-q-R | GGTACTCCAGAAGACCAGAGGA |
|  | *Tnf*-q-F | GCCTCTTCTCATTCCTGCTT |
|  | *Tnf*-q-R | CTCCTCCACTTGGTGGTTTG |
|  | *FasL*-q-F | GTATCAGCTCTTCCACCTGC |
|  | *FasL*-q-R | TGTTAAATGGGCCACACTCC |
|  | *Fas*-q-F | CAGACATGCTGTGGATCTGG |
|  | *Fas*-q-R | GTTGGCATGGTTGACAGCAA |
|  | *P53*-q-F | CTCTCCCCCGCAAAAGAAAAA |
|  | *P53*-q-R | CGGAACATCTCGAAGCGTTTA |
|  | *Casp3*-q-F | TGGTGATGAAGGGGTCATTTATG |
|  | *Casp3*-q-R | TTCGGCTTTCCAGTCAGACTC |
|  |  |  |
| Figure S4D & J | *Fasn*-F | GGAGGTGGTGATAGCCGGTAT |
|  | *Fasn*-R | TGGGTAATCCATAGAGCCCAG |
|  | *Hk2*-F | TGATCGCCTGCTTATTCACGG |
|  | *Hk2-*R | AACCGCCTAGAAATCTCCAGA |
|  | *Pkm*-F | GCCGCCTGGACATTGACTC |
|  | *Pkm*-R | CCATGAGAGAAATTCAGCCGAG |
|  | *Pfkm*-F | TGTGGTCCGAGTTGGTATCTT |
|  | *Pfkm*-R | GCACTTCCAATCACTGTGCC |
|  | *Insr-F* | ATGGGCTTCGGGAGAGGAT |
|  | *Insr-R* | GGATGTCCATACCAGGGCAC |
|  | *Igf1r*-F | GTGGGGGCTCGTGTTTCTC |
|  | *Igf1r*-R | GATCACCGTGCAGTTTTCCA |
|  | *Igf2r*-F | GGGAAGCTGTTGACTCCAAAA |
|  | *Igf2r*-R | GCAGCCCATAGTGGTGTTGAA |
|  | *Slc2a1*-F | CAGTTCGGCTATAACACTGGTG |
|  | *Slc2a1*-R | GCCCCCGACAGAGAAGATG |
|  | *Slc2a2*-F | TCAGAAGACAAGATCACCGGA |
|  | *Slc2a2*-R | GCTGGTGTGACTGTAAGTGGG |
|  | *Slc2a3*-F | ATGGGGACAACGAAGGTGAC |
|  | *Slc2a3*-R | GTCTCAGGTGCATTGATGACTC |
|  | *Slc2a4*-F | GTGACTGGAACACTGGTCCTA |
|  | *Slc2a4*-R | CCAGCCACGTTGCATTGTAG |
|  |  |  |
|  | *Gapdh*-F | AGGTCGGTGTGAACGGATTTG |
|  | *Gapdh*-R | TGTAGACCATGTAGTTGAGGTCA |
